# Supplementary material for: Anterior tooth-use behaviors among early modern humans and Neandertals
Source: PLoS One. 2019 Nov 27;14(11):e0224573. doi: 10.1371/journal.pone.0224573 (PMC6880970; doi:10.1371/journal.pone.0224573)
Supplement: S1 File — (DOCX) [file pone.0224573.s001.docx]

**S1. Supplemental information for each early modern human used here.**

**Site and Country:** Dolní Vĕstonice, Czech Republic

**Location:** Open-air sites (DV I and II) on the slopes of the Pavlov Hills; 48° 53' 0" N.

**Individual/s and tooth:** Specimen Tooth *epLsar* *Tfv*

3 (Klíma's DV III-1949): RI_1_ 0.0016 10577.27

13 LI^1^ 0.0018 12632.03

14 RI_1_ 0.0036 4848.98

15 RI^1^ 0.0055 6034.07

**Excavation context:** Dolní Vĕstonice I (DV I) underwent systematic excavations from 1924 to 1942 by K. Absolon and A. Bohmers. Systematic excavations did not resume until 1947 (and periodically thereafter until the late 1970s) under the direction of B. Klíma [1]. Dolní Vĕstonice II (DV II) was excavated first by B. Klíma in 1959-1960 and again, with J. Svoboda, from 1985-1991 [1]. Dolní Vĕstonice 3, an adult female, was located in Klíma's layer 9 [see 2, pp.139-140] by B. Klíma in July 1949 at DV I in the "upper part of the site at the margin of settlement unit 1" [1, p. 12]. She is estimated to be approximately 30-40 years old [3]. Dolní Vĕstonice 13-15 represents an all-male* triple burial, and was located in cultural layer 6 [4] in August 1986 at the "southern margin of the settlement concentration on the top of the Dolní Vĕstonice II site" [5]. All three individuals were approximately 20 years old [6].

**Environment:** Both sites: "...the landscape was partly covered by wooded areas (arboreal pollen usually exceeds 50%), dominated by conifers, and accompanied by deciduous trees, including a few more pretendous species such as oak, beach, and yew. ...we would reconstruct a variable and changing environment, both in terms of time and altitude on the landscape, that varies among steppe, shrub and steppe, and partially forested landscapes" [Summary of paleoenvironmental proxies from DV; 7, p. 6];

Generally cold conditions prevailed [charcoal analysis; 8];

DV I: "...the vegetation of that time was not monotonous, that it was not a mere steppe, but rather a forest-steppe and that it was also covered with small groves, maybe prevailingly in river valleys and at climatically more advantageous places" [fauna; 9, p. 196];

DV II: "We expect, therefore, that the cultural layer developed during a longer time-span, in the period of changing climatic oscillations in transition from the Würmian Interpleniglacial to the Upper Pleniglacial, and at the limit of various altitudinal zones. This climatical instability, or contact of various environments, is important for understanding the Upper Paleolithic adaptations during human occupation of the site" [sedimentology and palynology; 4, p.13);

DV II: "Steppe environment with heliophilous vegetation and islands of thermophilous deciduous and coniferous trees" [palynology from cultural layer; 10, p. 84];

DV II: "The fauna available demonstrates environmental conditions prevailing at the site when it was occupied by the Pavlovian people. This situation can be interpreted as a cold subarctic tundra, which is fully in agreement with the discovery of bones of mammoths and reindeer in the same horizon" [molluscs from the cultural layer; 12, p. 92];

DV II: "Continental and relatively cool, but not characterized by extremely low temperatures, permafrost, or tundra vegetation" [summary of paleoenvironmental reconstructions; 12];

DV II: At least partially forested; presence of yew suggests the site may have been slightly milder and more humid that previously thought [wood charcoal from DV II; 12].

**Date/s:** DV I: Interstadial Würm 2/3 [chronostratigraphy of layer 10; 2];

DV I: 25,950 +630/-580 BP [^14^C of charcoal from cultural layer in the upper part of the site (trench 10/90); 4];

DV I: early Middle Upper Paleolithic [archaeology; 3];

DV I: 22,840 ± 200 BP [uncalibrated radiocarbon from human bone of DV 35; 7];

DV II: 26,640 BP [uncalibrated radiocarbon of charcoal associated with DV 13-15; 7];

DV II: 31,305 - 30,996 BP [calibrated radiocarbon of charcoal associated with DV 13-15; 7, 8].

**Note/s: ***DV XV was originally reported to be a female [see 3 and 6, p. 834, including the footnote].

Presence of smaller carnivores in the faunal assemblages at DV II suggest "systematic fur and hide working was involved, and the use-wear analysis seems to confirm this hypothesis" [1, p. 13].

Also consulted: [13].

**Site and Country:** Pavlov I, Czech Republic

**Location:** Open-air site on the base of the Pavlov Hills; 48° 52' 0" N.

**Individual/s and tooth:** Specimen Tooth *epLsar* *Tfv*

1 RI^1^ 0.0022 13068.70

5/1 (site no. 519156) RI_1_ 0.0062 15591.58

23/1 (site no. i592256/m84) LI_1_ 0.0026 6899.53

25/1 (site no. 641456) RI_1_ 0.0020 4574.83

**Excavation context:** Pavlov I was systematically excavated from 1952 to 1965 and again from 1971 to 1972 under the direction of B. Klíma [1]. Although the site is divided into the northwestern and southeastern areas, the same radiocarbon dates were documented [1]. The male Pavlov I burial, was located in September 1957 in the northwestern area of the site "at the edge of the erosional furrow, under the bloc of undisturbed sediments separating the 1956 and 1957 areas" [5, 14, 15 p. 14, 16]. The isolated dental remains (5/1, 23/1, and 25/1) were located in the southeastern area, also in 1957 [5]. All the individuals used here were located by B. Klíma.

**Environment:** "The dominance of conifers suggests that the landscape was not a subarctic steppe, but in lower altitudes we may reconstruct a cold forest-steppe, in protected places accompanied by more pretenduous deciduous trees" [dendrochronology; 18, p. 178].

"...the vegetation of that time was not monotonous, that it was not a mere steppe, but rather a forest-steppe and that it was also covered with small groves, maybe prevailingly in river valleys and at climatically more advantageous places. ...the climate had a continental character, was certainly cold, but the presence of the above deciduous trees absolutely excludes extremely cold climate and/or permafrost. It is in accordance with my conclusions from the fauna" [fauna; 9, p. 196];

"...we incline to reconstruct the Pavlovian landscape during the Interpleniglacial as follow: small lakes, pools and swamps in the Dyje foodplain, gallery forests with poplar and willow (perhaps alder), loess cover on hill slopes colonised by conifer patches on exposed slopes, subalpine grass communities on the rocky summits" [charcoal; 18, p. 439].

"...the landscape was partly covered by wooded areas (arboreal pollen usually exceeds 50%), dominated by conifers, and accompanied by deciduous trees, including a few more pretendous species such as oak, beach, and yew. ...we would reconstruct a variable and changing environment, both in terms of time and altitude on the landscape, that varies among steppe, shrub and steppe, and partially forested landscapes" [Summary of paleoenvironmental proxies from DV; 7, p. 6.

**Date/s:** 25,675 +2750/-2045 BP [peat-bog at Bulhary, near Pavlov; 9];

26,730-25,020 BP [uncalibrated radiocarbon dates; 7].

**Note/s:** "Attrition of the permanent dentition [of Pavlov I] represents gradual loss of hard dental tissues due to use-wear of the individual teeth" [16, p. 75].

Also consulted: [13].

**Site and Country:** Brassempouy, France

**Location:** La Galerie des Hyènes, Brassempouy cave system; 43° 48' 0" N

**Individual/s and tooth:** Specimen Tooth *epLsar* *Tfv*

262 (aka BR 94) LI^2^ 0.0027 2061.43

1046 (aka BR 90 LI^1^ 0.0040 12077.54

**Excavation context:** The Brassempouy cave system was excavated by Dubalen in 1880, Laporterie and Dufour in 1890, and Piette and Laporterie from 1894-1897 [19-23]. Further excavations in la grotte des Hyènes and la galerie Dubalen were directed by H. Delporte and D. Buisson beginning in 1981 [23, 24]. Both teeth were found by Delport between 1981 and 1996 in la grotte des Hyènes [23]. Incisor no. 262 was found in level 2E, square BY10 and no. 1046 was found in level 2C, square BA10 [23].

**Environment:** "...les animaux représentés dans les couches aurignaciennes sont dans leur majorité des espèces adaptées à un environnement ouvert et froid...[fauna from ensemble 2 at la grotte des Hyènes; 22, p. 76];

Cold, open steppe [fauna from ensemble 2 at la grotte des Hyènes; 25].

**Date/s:** 35,000 - 30,000 BP [^14^C dating of bones from the Aurignacian layers; 23];

Level 2E: 31,960±160 - 26,870±500 (GifA 8570 excluded);

Levels 2A-2C: 32,190 ±620 - 31,690±780 [^14^C dating; 26].

**Note/s:** Taxonomic affinities of the isolated teeth cannot be confirmed [23]. As they are associated with the Aurignacian, they are considered to be early modern humans here. See 23, pp. 53-54 as a caution, however.

Tooth 1046: "Sur la face vestibulaire, il n'y a aucune strie due à un objet en silex" [23, p. 62].

**Site and Country:** Farincourt, France

**Location:** Les grottes d'Farincourt; 47° 45' 0" N

**Individual/s and tooth:** 1: RI_2_ *epLsar*: 0.0020 *Tfv*: 3556.82

**Excavation context:** Farincourt was excavated periodically throughout the 19th century, but it wasn't until the excavations directed by R. Joffroy and P. Mouton from 1933 until 1955 that systematic analysis occurred [27]. Joffry and Mouton began excavations on caves I and II, but located III in the late 1930s [27]. Farincourt I was located at the entrance of the cave. Although it was originally reported that the skeleton was found in an area without stratigraphy [“A l'entrée de la grotte III, dans un terrain meuble sans stratigraphie..." 28, p. 99], a revision was printed that indicated the remains came from level B ["Une mandibule et un humérus humains, très fossilisés", 29, p. 209]. This individual was determined to be a young female of approximately 20-25 years of age [29, 30].

**Environment:** Cold, open steppe; "L'habitat correspondant devait être d'ailleurs fort inconfortable" [fauna; 29, p. 209].

**Date/s:** "Une phase finale du Magdalénien" [28, p. 100];

Magdalenian III [archaeology; 29].

**Note/s:**  Farincourt I is sometimes referred to as Farincourt III, 1 (mandible) and Farincourt III, 2 (humerus). [See 29, p. 218 and 30, p. 7].

**Site and Country:** Isturitz, France

**Location:** La grotte d’Isturitz; 43° 20' 1" N

**Individual/s and tooth:** Ist 115 (aka Ist III 1937): RI_2_ *epLsar*: 0.0032 *Tfv*: 12411.73

**Excavation context:** Passemard [31] described two parallel galleries (Galerie Nord and Galerie Sud, named for the direction their entrances faced) at Isturitz, where he excavated from 1912 until 1922. Saint-Périer then excavated from 1928 until 1948 [32]. The cave was divided into the Salle d'Isturitz (north side, also called Grande Salle) and Salle de St. Martin (south side). See Passemard [31, p. 9] and Saint-Périer and Saint Périer [32, p. 5] for exact dividing lines for each gallery. Ist 115* was found in level III, the Aurignacian final level, and is estimated to be a young adult male of approximately 20-30 years old [32, but see 33].

**Environment:** Forest-steppe; "L'existence certaine d'os de Saïga à Lespugue et à Isturitz montre, au contraire, que les steppes n'étaient pas éloignés et que la faune des forêts, essentiellement représentée par *Cervus elaphus* dans les deux gisements, voisinait avec la faune steppique" [fauna; 32, p. 73];

Cold, dry steppe; "Les résultats donnés par cet échantillon sont très proches des précédents: climat très froid, très sec...Nous donc là un type de steppe qui s'établit à l'extrême fin du gravettien, se développe à travers le solutréen et la première partie du magdalénien, malgré quelques flutuations dans la température et les degrés d'humidité" [palynology of the Aurignacian final level; 34, p. 623].

**Date/s:** Aurgicnacian final [archaeology; 32].

**Note/s: ***Ist 115 is also known as the mandible Ist III 1937 or as "Mandibule a" as described by Saint-Périer and Saint-Périer [32, p. 75-76; 33]. This site has been reconstructed as mixed and open habitats. In an abundance of caution, it has been assigned to the "mixed" habitat category.

**Site and Country:** Lachaud, France

**Location:** Grottes du Pouget; 45° 07' 0" N

**Individual/s and tooth:** Specimen Tooth *epLsar* *Tfv*

Ferembach’s 4* (aka 1980-7-4) isolated I_2_ 0.0055 2165.20

Ferembach’s 5* (aka 1980-8-1) LI_1_ 0.0052 6438.89

**Excavation context:** The grottes du Pouget (or Pouzet) were found during road construction in 1866, and underwent informal surveys by several amateur excavators [35]. It wasn't until the systematic excavations directed by A. Cheynier in the 1940s that l'abri Lachaud (or Pouget 3) was located and examined [35]. Both individuals used here were found in the proto-Magdalenian II layer [35]. Lachaud 4 is estimated to be a teenager, whereas Lachaud 5 is estimated to be approximately 13-14 years old [36].

**Environment:** Open steppe [fauna; 37].

**Date/s:** Proto-Magdalenian II [archaeology; 37].

**Note/s:** *Lachaud 4 is an individual represented by two isolated lateral incisors; Lachaud 5 is also known in the literature as "mandibule A" [36].

**Site and Country:** Les Rois, France

**Location:** Les Rois rock shelter; 45° 35' 0" N

**Individual/s and tooth:**

Specimen Tooth *epLsar* *Tfv*

**Based on Vallois, 1958:146-148:**

R.50, #45, No. 1 *incisives supérieurs* LI^1^ 0.0035 7315.66

Unnumbered, but described as No. 3 *incisives supérieurs* LI^1^ 0.0031 16735.67

Unnumbered, but described as No. 7 *incisives inférieurs* LI_1_ 0.0022 14808.91

R.51 #17, No. 5 *incisives inférieurs* LI_2_ 0.0043 11631.48

Ramirez Rozzi et al. [38]: #35 LI^2^ 0.0037 12704.37

**Excavation context:** Les Rois cave was found in the late 1920s, and test pits were dug near the entrance during the 1930s by an amateur excavator; however, it wasn't until Mouton and Joffroy's systematic excavations from 1948 to 1954 that three archaeological units (B, A1, and A2) were recognized [38, 39]. All isolated teeth came from subunit A2β [38].

**Environment:** Cool, open steppe; "Au point de vue du climat, le gisement des rois vient donc confirmer ce qu'on savait déjà: Aurignacien I très froid et très sec, périodes suivantes d'autant moins rudes et moins sèches qu'on s'approches de l'Aurignacien final" [fauna; 40, p. 28].

**Date/s:** 30,250 ± 220 - 27,790 +200/-190 BP [AMS ^14^C; 38].

**Site and Country:** Rond-du-Barry, France

**Location:** La grotte du Rond du Barry; 44° 47' 0" N

**Individual/s and tooth:** Le crâne du Rond-du-Barry: RI^2^ *epLsar*: 0.0025 *Tfv*: 12740.69

**Excavation context:** Systematic excavations were carried out under the direction of R. de Bayle des Hermens beginning in 1966 until 1988. In 1969, level F was reached and then divided into three sub-units: F1, F2, and F3 [41]. The cranium (perhaps a secondary burial) was found in July 1986 in the northwest area of the cave, and is estimated to be a male of approximately 50+ years old [42, 43].

**Environment:** Cold, open steppe; "Les résultats confirment les observations des années précédentes, c'est-à-dire le caractère froid arctique des couches F et E..." [fauna; Chaline in 41, p. 35].

**Date/s:** Magdalenian I, [archaeology; 41, 44];

17,100 ± 450 BP [^14^C of bone; 42];

Magdalenian or Badegoulien [based on different ^14^C dates from different sectors of the cave where level F2 is found; 44].

**Site and Country:** Saint-Germain-la-Rivière, France

**Location:** Saint-Germain-la-Rivière rock shelter; 44° 57' 0" N

**Individual/s and tooth:** 1970-7-12: LI^1^ *epLsar*: 0.0019 *Tfv*: 11514.50

**Excavation context:** Saint-Germain-la-Rivière was first discovered in 1928, and was excavated by several individuals throughout the 20th century, including Mirande and Lépront (1929-1933), Blanchard (1933-1934), Trécolle (1960s), and Lenoir (1996) [45]. There are two rock shelters, Grand Abri and the Shelter of the Upper Terrace, which are separated by an area known as the Intermediate Talus [45]. The isolated tooth used here was excavated by Blanchard in the abri de la terrasse inférieure in layer A or B, attributed to the Magdalénien II and III, respectively [Vallois in 46]. Although its exact provenience is unknown, Vallois [46, p. 110] notes "...il paraît tout à fait plausible qu'ils datent bien de l'époque magdalénienne."

**Environment:** Open steppe [fauna of layers A and B; 46];

Steppe, pastureland [bone tools; Peyrony in 46].

**Date/s:** Magdalenian ["La totalité des restes humains de ce site provient incontestablement des couches magdaléniennes"; 47, p. 18];

15,780 ± 200 BP [^14^C of rib bone from the burial STG 4 (aka STG 1); 45];

16,890 ± 130 - 14,100 ± 160 BP [range of ^14^C available at all excavation areas of STG; 48].

**Site and Country:** Grimaldi Caves, Italy

**Location:** Grotte des Enfants (aka Grotta dei Fanciulli); 43° 47' 0" N

**Individual/s and tooth:** GdE 4 (burial III): LI^1^ *epLsar*: 0.0044 *Tfv*: 4151.13

**Excavation context:** Le grotte des Enfants is one of several caves on the French/Italy border, and was described as early as the 18th century; it was first excavated by E. Rivière in the 1870s and again by L. de Villeneuve beginning in 1901 [49]. de Villeneuve documented layers L through A, locating Grotte de Enfants 4 (burial III) in 1901, at the base of *foyer* H, at a depth of 7.05 m [50]. This individual was estimated to be an adult male [51].

**Environment:** Cold, open steppe; "Le climat était certainement froid"; [fauna of level H; 52, p. 19].

**Date/s:** Aurignacian [archaeology of tools associated with GdE 4; 52];

Final Gravettian [chronostratigraphy*; 53, p. 264];

Gravettian [lithics; 54, p. 121];

≈23,400 ± 190 – 24,800 ± 800 BP (AMS ^14^C date of contemporaneous Arene Candide 1 and Barma Grande 6, respectively [55].

**Note/s:** Formicola and Repetto [51, p. 60] note this about GdE 4 teeth: "The occlusal surface of the mandibular incisors exhibits a clear labial rounding, probably the effect of task activity carried out with anterior teeth. This conclusion is supported by microwear analysis. The presence of grooves and of many large pits and microflakes in anterior dentition is indicative of friction and power-grasping during non-alimentary tooth use."

*Palma di Cesnola [53, p. 264] refers to this individual as Enfants III, referring to it using the burial number, not the individual number. The description of its location and illustration clarifies that Palma di Cesnola [53] is discussing the same individual that was analyzed here.

**Site and Country:** Ohalo II, Israel

**Location:** Fisher-hunter-gatherer camp on the western shore of the Sea of Galilee; 32° 43' 0" N

**Individual/s and tooth:** Ohalo II H2: LI^1^ *epLsar*: 0.0025 *Tfv*: 14355.56

**Excavation context:** The site of Ohalo II was located in 1989 when the Sea of Galilee reached an extremely low level; a salvage excavation was then conducted by the Israel Antiquities Authority under the direction of D. Nadel [56]. Based on flora and faunal assemblages, Ohalo II was occupied for more than 6 months at a time, most likely during autumn through spring [56-58]. In 1991, a 35-40-year-old male was recovered from a shallow pit in a flexed position just west of Locus 5; as it "was firmly embedded in a hard gray sediment which proved to be difficult to remove in the field" [59, p. 217], it was removed in the laboratory [60].

**Environment:** "It is generally agreed that during the Last Glacial Maximum, when the site was occupied, the climate in the Levant was drier and probably colder than today” [56, p. 57];

Warm and mixed; Mixed environment of grassland, marsh, woodland, desert, and aquatic habitats. "The climate of Ohalo II was for the most part Mediterranean and the diversity of plant species was very similar to those of the present day. ...What is interesting to note, however, is the great variety of habitats illustrated by the avian species. ...This habitat distribution is not signficantly different, either in type of habitat or in its division of the landscape, from that of the region surrounding the Sea of Galilee in the present day. All these microenvironments are readily accessible within easy walking distance, or a day's walk (the more extreme arid/desert region), of the site" [avifauna; 58, pp. 87-88];

"These species reflect a Mediterranean park forest and grassland habitats" [fauna; 61, p. 24];

“Distinct habitats form the environment around the site: park-like forest, river-bank, saline, lake-habitat, and dry habitat…The large number of herbaceous and grass species suggests that the herbaceous plants grew in open areas of this park-like forest, which possibly extended onto the adjacent hill-slopes and near the lake. The main reason for this spatial reconstruction of this landscape is the relatively cold and dry climate of the LGM, which probably affected the vegetation and created a landscape similar to that of the Irano-Anatolian region” [plant assemblage; 62, p. S128];

"More than 100 taxa have been identified, and the species represent at least three habitats: bank/shore vegetation, a wide saline near the site, and an open park-forest with many grasses further away" [seed and wood analysis; 63].

**Note/s:** The skeleton is also referred to as H1, and the isolated mandible as H2 [see 64].

**Date/s:** ≈19,000 BP [^14^C of charcoal; 60];

≈22,500 - 23,500 BP [calibrated ^14^C; 65].

**Note/s:** A grinding stone, made of basalt, was located in hut 1. This, along with starch grains recovered from the stone, indicates grains were processed into flour or meal [66, 67]. This suggests abrasive loads may have been high for the individuals at Ohalo II.

**Site and Country:** Qafzeh, Israel

**Location:** Qafzeh cave (Jebel Qafzeh); 32° 41' 0" N

**Individual/s and tooth:** Specimen Tooth *epLsar* *Tfv*

5 RI^1^ 0.0038 10052.73

6 RI^1^ 0.0029 11922.77

7 LI^1^ 0.0041 0.00

9 LC^1^ 0.0045 10628.36

**Excavation context:** Qafzeh cave was first excavated by R. Neuville and M. Stekelis in 1934, and then continued by B. Vandermeersch from July 1965 until the summer of 1979 [see 68 for a delightful review]. While Neuville and Stekelis recognized layers A-M, Vandermeersch documented his layers as roman numerals I-XXIV [69, 70]. Qafzeh 5-7 were discovered by Neuville and Stekelis in 1934 and 1935 in layer L (Levalloisien inférieur). "...Q5 (Qafzeh 5) ont été trouvés par Stekelis contre la paroi Nord-Est du sondage de la terrasse" [70, p. 30). "...Q6 (Qafzeh 6) ont été trouvés dans la moitié Ouest du vestibule...au pied du seuil rocheux" [70, p. 30]. "Q7 (Qafzeh 7) a été découvert au cours de la campagne de 1935, tout contre Q6, en dégageant l'emplacement présumé des os des membres de ce dernier" [70, p. 32]. Qafzeh 9 was located by B. Vandermeersch in 1967 in layer XVII (Levalloiso-Mousterian) in the vestibule [70, 71]. Vandermeersch suggests that his layer XVII corresponds to Neuville's layer L, making these individuals contemporaneous [70, 72]. All individuals used here are adults; Qafzeh 6 and 9 are estimated to be a male and female, respectively, and Qafzeh 5 and 7 are of indeterminate sex [70, 72].

**Environment:** "Jusqu'au glaciaire würmien, c'est-à-dire à peu près jusqu'a la fin du Moustérien, un climat subtropical et sec a alterné à plusieurs reprises avec un climat chaud et humide, parfois nettement tropical" [sedimentology; 73, p. 239];

"The Mousterian fauna, fairly rich in Cervidae, Bovinae, but very poor in Gazelles proves that the climate cooler and moister than now" [fauna; 74, p. 87];

Warm and mixed; "The faunal assemblage of Qafzeh is predominated by open-country, steppe or savanna species...There is a strong Afro-Arabian stamp upon the fauna of Qafzeh, indicating savanna conditions during this period" [microfauna; 75, p. 11];

"...throughout the time of occupation (MIS 5d-a) the Qafzeh Cave region was more open than today...Our inference of drier conditions is consistent with the recent study of the microvertebrate remains from the lower unit... [δ^13^C from goats; 76, p. 70].

**Date/s:** 92,000 ± 5,000 BP [thermoluminescence of burnt flint from the hominin-bearing layers; 77];

96,000 ± 13,000 (early uptake) and 115,000 ± 15,000 (linear uptake) [ESR of large mammal teeth from the hominin-bearing layers XV-XXI; 78].

**Site and Country:** Es-Skhūl, Israel

**Location:** Mugharet es-Skhūl rock shelter; 32° 37' 0" N

**Individual/s and tooth:** Specimen Tooth *epLsar* *Tfv*

II LI_1_ 0.0023 13033.95

IV LI^1^ 0.0013 5765.14

V RI^1^ 0.0020 15303.62

**Excavation context:** The es-Skhul rockshelter was excavated by T.D. McCown in 1931 and 1932 after a small trial excavation was conducted in 1929 [79]. McCown [79] identified three main archaeological units, which he labeled A, B, and C; all the human remains were found in 1932 in area D, layer B, which is attributed to the Levalloiso-Mousterian. While layer B is divided into B^1^ and B^2^, no lithic or faunal differences were found between these two sub-units; they were differentiated by soil matrix composition and flint patina color [see 79, p. 96]. Skhul II is estimated to be a female and approximately 30-40 years old, Skhul IV is a male aged 40-50 years, and Skhul V is also a male between 30-40 years old [80].

**Environment:** Warm and mixed; "Approach of wet conditions at close. Climate warm and dry, with permanent rivers" [fauna; 81, p. 157];

**Date/s:** 81,000 ± 15,000 (early uptake) - 101,000 ± 12,000 BP (linear uptake) [82];

119,000 ± 18,000 BP [thermoluminescence; 83];

116,000 +43,000/-24,000 [combined U-series/ESR dating of Skhul II; 84];

93,000 +21,000/-12,000 BP [combined U-series/ESR dating of a pig tooth associated with Skhul V; 84].

**References Cited**

1. Svoboda JA. The archaeological contexts of the human remains. In: Trinkaus E, Svoboda J, editors. Early Modern Human Evolution in Central Europe. New York: Oxford University Press; 2006. pp. 9-14.

2. Klíma B. Dolní Vĕstonice: Výzkum tábořiště lovců mamutů v letech 1947-1952. Praha: Nakladatelství Československé Adademie Věd; 1963.

3. Sládek V, Trinkaus E, Hillson SW, Holliday TW. The People of the Pavlovian. Skeletal Catalogue and Osteometrics of the Gravettian Fossil Hominids from Dolní Věstonice and Pavlov. Brno: Vydal Archeologický ústav Akademie věd České republiky; 2000.

4. Svoboda J. Dolní Vĕstonice II - Western Slope. Liège : Etudes et Recherches Archéologiques de l'Université de Liège, no. 54; 1991. pp. 5-60.

5. Svoboda JA. The burials: ritual and taphonomy. In: Trinkaus E, Svoboda J, editors. Early Modern Human Evolution in Central Europe. New York: Oxford University Press; 2006. pp. 15-26.

6. Klíma B. A triple burial from the Upper Paleolithic of Dolní Vĕstonice, Czechoslovakia. J Hum Evol. 1988; 16(7-8): 831-835. doi: 10.1016/0047-2484(87)90027-3.

7. Svoboda JA. The archaeological framework. In: Trinkaus E, Svoboda J, editors. Early Modern Human Evolution in Central Europe. New York: Oxford University Press; 2006. pp. 6-8.

8. Beresford-Jones D, Taylor S, Paine C, Pryor A, Svoboda J, Jones M. Rapid climate change in the Upper Paleolithic: the record of charcoal conifer rings from the Gravettian site of Dolní Věstonice, Czech Republic. Quatern Sci Rev. 2011; 30(15): 1948-1964. doi: 10.1016/j.quascirev.2011.04.021.

9. Musil R. The Fauna. In: Svoboda J, editor. Pavlov I: Excavations 1952-1953. Liège: Service de préhistoire, Université de Liège; 1994. pp. 181-209.

10. Svobodová H. The pollen analysis of Dolní Věstonice II, section no. 1. In: Svoboda J, editor. Dolní Věstonice II: Western Slope. Liège: Etudes et Recherches Archéologiques de l'Université de Liège, no. 54; 1991. pp. 75-88.

11. Kovanda J. Molluscs from the section with the skeleton of Upper Paleolithic man at Dolní Vĕstonice. In: Svoboda J, editor. Dolní Věstonice II: Western Slope. Liège: Etudes et Recherches Archéologiques de l'Université de Liège, no. 54; 1991. pp. 89-96.

12. Mason SLR, Hather JG, Hillman GC. Preliminary investigation of the plant macro-remains from Dolní Věstonice II, and its implications for the role of plant foods in Palaeolithic and Mesolithic Europe. Antiquity. 1994; 68(258): 48-57. doi: 10.1017/S0003598X00046184.

13. Hillson SW. Dental morphology, proportions, and attrition. In: Trinkaus E, Svoboda J, editors. Early Modern Human Evolution in Central Europe. New York: Oxford University Press; 2006. pp. 179-223.

14. Klíma B. Objev Paleolitického pohřbu v Pavlovĕ. Archeologické rozhledy. 1959; 11: 305-316.

15. Klíma B. Bone Industry, Decorative Objects and art. In: Svoboda J, editor. Pavlov I - Northwest: the Upper Paleolithic Burial and its Settlement Context. Academy of Sciences of the Czech Republic. Brno: Institute of Archaeology; 1997. pp. 227–286.

16. Vlček E. Human remains from Pavlov and the biological anthropology of the Gravettian human population of South Moravia. In: Svoboda J, editor. Pavlov I - Northwest: the Upper Paleolithic Burial and its Settlement Context. Academy of Sciences of the Czech Republic. Brno: Institute of Archaeology; 1997. pp. 53-153.

17. Opravil E. The vegetation. In: Svoboda J, editor. Pavlov I: Excavations 1952-1953. Service de préhistoire. Liège: Université de Liège; 1994. pp. 176-179.

18. Damblon F. Anthracology. In: Svoboda J, editor. Pavlov I - Northwest: the Upper Paleolithic Burial and its Settlement Context. Academy of Sciences of the Czech Republic. Brno: Institute of Archaeology; 1997. pp. 437-442.

19. Piette E. Fouilles faites à Brassempouy en 1895. Bull Soc Anthropol Paris. 1895; 6: 659-663.

20. Piette E and Laporterie J. Fouilles a Brassempouy en 1896. L'Anthropologie. 1897; 8: 165-173.

21. Piette E and Laporterie J. Fouilles a Brassempouy en 1897. L'Anthrpologie. 1898; 9: 531-555.

22. Bon F. L'Aurignacien entre Mer et Océan. Réflexion sur l'unité des phases anciennes de l'Aurignacien dans le sud de la France, Mémoire XXIV. Paris: Société Préhistorique Française; 2002.

23. Henry-Gambier D, Maureille B, White R. Vestiges humains des niveaux de l'Aurignacien ancien du site de Brassempouy (Landes). Bull Mém Soc Anthropol Paris. 2004; 16(1-2): 49-87.

24. Delporte H and Buisson D. Brassempouy: les fouilles de 1988 à 1990. Bull Soc Borda. 1991; 422: 143-157.

25. Letourneux C. Etude taphonomique et archeozoologique des niveaux Aurignaciens anciens de la grotte des Hyènes (Brassempouy, Landes) "Qui a mange quoi?" Archéologie des Pyrénées Occidentales et des Landes. 2005; 24: 85-102.

26. White R. Systems of personal ornamentation in the early Upper Paleolithic: methodological challenges and new observations. In: Bar Yosef B, Boyle K, Mellars P, Stringer C, editors. Rethinking the Human Revolution: New Behavioural and Biological Perspectives on the Origin and Dispersal of Modern Humans. Cambridge: McDonald Institute for Archaeological Research; 2007. pp. 287-302.

27. Pernot P and Costa L. La grotte III de Farincourt (Haute-Marne): nouvelles recherches. Préhist Protohist Champagne-Ardenne. 1995; 19: 27-39.

28. Joffroy R and Mouton P. La station magdalénienne de Farincourt (H.-M.). Bull Soc Préhist Fr. 1946; 43(3-4): 91-100. doi: 10.3406/bspf.1946.2449.

29. Mouton P, Joffroy R, Sauter MR. Précisions nouvelles sur les stations magdaléniennes de Farincourt (Haute-Marne). Rev Archeol Est. 1956; 7: 193-223.

30. Sauter MR. Etude des vestiges osseux humains des grottes préhistoriques de Farincourt (Haute-Marne, France). Arch Suisses Anthropol Gen. 1957; 22(1): 6-37.

31. Passemard E. La caverne d'Isturitz en Pays Basque. Préhistoire. 1944; 9: 7-95.

32. Saint-Périer R and Saint-Périer S. La grotte d’Isturitz III: Les Solutréens, les Aurignaciens et les Moustériens. Paris: Archives de I’Institut de Paléontologie Humaine 24; 1952.

33. Gambier D. Les vestiges humains du gisement d'Isturitz (Pyrénées-Atlantiques). Études anthropologique et analyse des traces d'action humaine intenionnelle. Antiquités Nationales. 1990; 22/23: 9-26.

34. Leroi-Gourhan A. Résultats de l'analyse pollinique de la grotte d'Isturitz. Bull Soc Préhist Fr. 1959; 56(9): 619-624. doi: 10.3406/bspf.1959.3615.

35. Cheynier A. L'abri Lachaud a Terrasson (Dordogne), Préhistoire, Tome XVI. Paris: Presses Universitaries de France; 1965.

36. Ferembach D. Les restes humains de l'abri Lachaud. Bull Mém Soc Anthropol Paris. 1957; 8(1): 61-80.

37. Cheynier A. Stratigraphie de l'abri Lachaud et les cultures des bords abattus. Arch Préhist Levantina. 1953; 4: 25-55.

38. Ramirez Rozzi FV, d'Errico F, Vanhaeren M, Grootes PM, Kerautret B, Dujardin, V. Cutmarked human remains bearing Neandertal features and modern human remains associated with the Aurignacian at Les Rois. J Anthropol Sci. 2009; 87: 153-185. pmid: 19663173.

39. Vallois HV. Les restes humains d'age Aurignacian de la grotte des Rois, Charente. Bull Mém Soc Anthropol Paris. 1958; 9(4): 138-159. doi: 10.3406/bmsap.1958.2717.

40. Mouton P and Bouchud J. Le matériel osseux - techniques de chasse et de dépecage. In: Mouton P, editor. Le Gisement Aurgicancien des Rois a Mouthiers (Charente). Paris: Centre National de la Recherche Scientifique; 1958. pp. 25-31.

41. Bayle des Hermens R (de). Note préliminaire sur le Magdalénien ancien de la couche F2 de la grotte du Rond-du-Barry. L'Anthropologie. 1974; 78(1): 17-36.

42. Bayle des Hermens R (de) and Heim J-L. Découverte d'un crâne humain dans une sépulture secondaire du Magdalénien I de la grotte du Rond-du-Barry, Polignac, Haute-Loire. C R Acad Sci Paris 1989; 309(Série II): 1349-1352.

43. Heim J-L. Le crâne magdalénien du Rond-du-Barry (Haute-Loire). Documents préhistoiriques. 1992; 2: 53-61.

44. Rémy D. Caractérisation techno-économique d'industries en bois de cervidés du Badegoulien et du Magdalénien: le cas du Rond-du-Barry (Haute-Loire) et de Rochereil (Dordogne). PhD dissertation, Université Paul Valéry - Montpellier. 2013.

45. Vanhaeren M, d'Errico F. Grave goods from the Saint-Germain-la-Rivière burial: evidence for social inequality in the Upper Paleolithic. J Anthropol Archaeol. 2005; 24(2): 117-134. doi: 10.1016/j.jaa.2005.01.001.

46. Blanchard R, Peyrony D, Vallois H-V. Le gisement et le squelette de Saint-Germain-la-Rivière. Archives de l'Institut de Paléontologie Humaine, Mémoire 34. Paris: Masson; 1972.

47. Gambier D and Lenoir M. Les vestiges humains du Paleolithique superieur en Gironde. Bull Soc Anthropol S O. 1991; 26(1): 2-31.

48. Gambier D, Valladas H, Tisnérat-Laborde N, Arnold M, Bresson F. Datation de vestiges humains présumés du Paléolithique supérieur par la méthod du carbone 14 en spectrométrie de masse par accélérateur. Paleo. 2000; 12: 201-212. doi: 10.3406/pal.2000.1602.

49. Villeneuve L (de). Les Grottes de Grimaldi (Baoussé-Roussé): Historique et Description. Monaco: Imprimerie de Monaco; 1906.

50. Verneau R. Les Grottes de Grimaldi (Baoussé-Roussé): Anthropologie. Monaco: Imprimerie de Monaco; 1906.

51. Formicola V and Repetto E. The dentition of the "Cro-Magnon type" Grotte des Enfants 4 (Grimaldi, Italy). B Mus Anthropol Préhist Monaco. 1989; 32: 51-52.

52. Lacorre F and Barral L. Aperçus nouveaux sur les industries et les ages des squelettes de la grotte des Enfants à Grimaldi. Riv studi liguri. 1948; 14: 5-38.

53. Palma di Cesnola A. Le Paléolithique Supérieur en Italie. Grenoble: Jérôme Millon; 2001.

54. Gambier D. La Sépulture des Enfants de Grimaldi (Baoussé-Roussé, Italie). Anthropologie et Palethnologie Funéraire des Populations de la Fin du Paléolithique Supérieur. Paris: CTHS, Réunion des musées nationaux; 2001.

55. Formicola V. Grimaldi, Grotte des Enfants 1901. J Anthropol Sci. 2005; S83: 77-79.

56. Nadel D. Ohalo II - a preliminary report. J Israel Prehist Soc. 1990; 23: 48-59.

57. Nadel D. Ohalo II - the third season. J Israel Prehist Soc. 1991; 24: 158-163.

58. Simmons T, and Nadel D. The avifauna of the Early Epipalaeolithic site of Ohalo II (19,400 years BP), Israel: species diversity, habitat and seasonality. Int J Osteoarchaeol. 1998; 8(2): 79-96. doi: 10.1002/(SICI)1099-1212(199803/04)8:23.0.CO;2-I.

59. Hershkovitz I, Speirs MS, Frayer D, Nadel D, Wish-Baratz S, Arensburg B. Ohalo II H2: A 19,000-year-old skeleton from a water-logged site at the Sea of Galilee, Israel. Am J Phys Anthropol. 1995; 96(3): 215-234. doi: 10.1002/ajpa.1330960302.

60. Nadel D, Carmi I, Segal D. Radiocarbon dating of Ohalo II: archaeological and methodological implications. J Archaeol Sci. 1995; 22(6): 811-822. doi: 10.1016/0305-4403(95)90010-1.

61. Rabinovich R. The mammal bones: environment, food and tools. In: Nadel D, editor. Ohalo II, a 23,000-year-old Fisher-Hunter-Gatherers' Camp on the Shore of the Sea of Galilee. Haifa: Hecht Museum and University of Haifa; 2002. pp. 24-27.

62. Weiss E, Kislev ME, Simchoni O, Nadel D. Small-grained wild grasses as staple food at the 23,000-year-old site of Ohalo II, Israel. Econ Bot. 2004; 58(1): S125-S134. doi: 10.1663/0013-0001(2004)58[S125:SWGASF]2.0.CO;2.

63. Nadel D, Grinberg U, Boaretto E, Werker E. Wooden objects from Ohalo II (23,000 cal BP), Jordan Valley, Israel. J Hum Evol. 2006; 50(6): 644-662. doi: 10.1016/j.jhevol.2005.12.010.

64. Mahoney P. Human dental microwear from Ohalo II (22,500-23,500 cal BP), Southern Levant. Am J Phys Anthropol. 2007; 132(4): 489-500. doi: 10.1002/ajpa.20548.

65. Nadel D, Tsatskin A, Belmaker M, Kislev ME, Rabinovich R, Weiss U, et al. J Israel Prehist Soc. 2002; 32: 17-48.

66. Piperno DR, Weiss E, Holst I, Nadel D. Processing of wild cereal grains in the Upper Palaeolithic revealed by starch grain analysis. Nature. 2004; 430(7000): 670-673. doi: 10.1038/nature02734.

67. Weiss E, Kislev ME, Simchoni O, Nadel D. Tschauner, H. Plant-food preparation area on an Upper Paleolithic brush hut floor at Ohalo II, Israel. J Archaeol Sci. 2008; 35(8): 2400-2414. doi: 10.1016/j.jas.2008.03.012.

68. Vandermeersch B. The excavation of Qafzeh: it's contribution to knowledge of the Mousterian in the Levant. Bull Cent Rech Fr Jer. 2002; 10: 65-70.

69. Neuville R. Le Paléolithique et le Mésolithique dans le désert de Judée. Arch l’Inst Paléont Hum. Paris: Masson, 270; 1951.

70. Vandermeersch B. Les Hommes Fossiles de Qafzeh (Israël). Paris: Éditions du Centre National de la Recherche Scientifique; 1981.

71. Neuville R. Jebel Qafzeh, Excavations in Palestine, 1934-5. Q Depart Antiq Palestine. 1936; 5: 199.

72. Vallois HW, Vandermeersch B. The Mousterian skull of Qafzeh (Homo VI). An anthropological study. J Hum Evol. 1975; 4(6): 445-455. doi: 10.1016/0047-2484(75)90142-6.

73. Neuville R. Le préhistorique de Palestine. Rev Biblique. 1934; 43(2): 237-259.

74. Bouchud J. Etude préliminaire de la faune provenant de la grotte du Djebel Qafzeh près de Nazareth (Israel). Paléorient. 1974; 2(1): 87-102.

75. Rabinovich R and Tchernov E. Chronological, paleoecological and taphonomical aspects of the Middle Paleolithic site of Qafzeh, Israel. In: Buitenhuis H and Uerpmann H-P, editors. Archaeozoology of the near East: Part II: Proceedings of the Second International Symposium on the Archaeozoology of Southwestern Asia and Adjacent Areas. Leiden: Backhuys Publishers; 1995. pp. 5-44.

76. Hallin KA, Schoeninger MJ, Schwarcz HP. Paleoclimate during Neandertal and anatomically modern human occupation at Amud and Qafzeh, Israel: the stable isotope data. J Hum Evol. 2012; 62(1): 59-73. doi: 10.1016/j.jhevol.2011.09.005.

77. Valladas H, Reyss JL, Joron JL, Valladas G, Bar-Yosef O, Vandermeersch B. Thermoluminescence dating of Mousterian Troto-Cro-Magnon remains from Israel and the origin of modern man. Nature. 1988; 331(6157): 614-616. doi: 10.1038/331614a0.

78. Schwarcz HP, Grün R, Vandermeersch B, Bar-Yosef O, Valladas H, Tchernov E. ESR dates for the hominid burial site of Qafzeh in Israel. J Hum Evol. 1988; 17(8): 733-737. doi: 10.1016/0047-2484(88)90063-2.

79. McCown TD. Chapter VI: Mugharet Es-Skhūl. Description and excavations. In: Garrod DAE and Bate DMA, editors. The Stone Age of Mount Carmel. Excavations at the Wady El-Mughara, Vol. I. Oxford: Clarendon Press; 1937. pp. 91-107.

80. McCown TD and Keith Sir A. The Stone Age of Mount Carmel. The Fossil Human Remains from the Levalloiso-Mousterian, Vol. II. Oxford: Clarendon Press; 1939.

81. Bate DMA. Description of faunal assemblages and their climatic inferences. In: Garrod DAE and Bate DMA, editors. The Stone Age of Mount Carmel. Excavations at the Wady El-Mughara, Vol. I. Oxford: Clarendon Press; 1937. pp. 143-157.

82. Stringer CB, Grün R, Schwarcz HP, Goldberg P. ESR dates for the hominind burial site of Es Skhul in Israel. Nature. 1989; 338(6218): 756-758. doi: 10.1038/338756a0.

83. Mercier N, Valladas H, Bar-Yosef O, Vandermeersch B, Stringer C, Joron J-L. Thermoluminescence date for the Mousterian burial site of Es-Skhul, Mt. Carmel. J Archaeol Sci. 1993; 20(2): 169-174. doi: 10.1006/jasc.1993.1012.

84. Grün R, Stringer C, McDermott F, Nathan R, Porat N, Robertson S, et al. U-series and ESR analyses of bones and teeth relating to the human burials from Skhul. J Hum Evol. 2005; 49(3): 316-334. doi: 10.1016/j.jhevol.2005.04.006.
