# Supplementary material for: Anterior tooth-use behaviors among early modern humans and Neandertals
Source: PLoS One. 2019 Nov 27;14(11):e0224573. doi: 10.1371/journal.pone.0224573 (PMC6880970; doi:10.1371/journal.pone.0224573)
Supplement: S2 File — (HTML) [file pone.0224573.s002.html]

Supplemental Materials


# Supplemental Materials

#### *Gregory J. Matthews, Ph.D.*

#### *June 3, 2019*

```
#############################################
#Read in the data from both neandertals and early modern humans
#############################################
neand <- read.csv("/Users/gregorymatthews/Dropbox/kruegerTeeth/neandertals2017.csv")
emh <- read.csv("/Users/gregorymatthews/Dropbox/kruegerTeeth/earlyModernHumans2017.csv")[,1:7]

neand$type <- "Neandertals"
emh$type <- "EMH"

#Create one combined data set
dat <- rbind(neand,emh)
names(dat) <- c("site","specimen","epLsar","Tfv","vegetation","location","time","type")

#Simple summaries of the data
summary(dat)
```

```
##              site      specimen             epLsar              Tfv       
##  Krapina       :10   Length:75          Min.   :0.000500   Min.   :    0  
##  LesRois       : 5   Class :character   1st Qu.:0.002125   1st Qu.: 6372  
##  Vindija       : 4   Mode  :character   Median :0.002800   Median :11041  
##  DolniVestonice: 4                      Mean   :0.003127   Mean   : 9879  
##  Pavlov        : 4                      3rd Qu.:0.003974   3rd Qu.:13054  
##  Qafzeh        : 4                      Max.   :0.006800   Max.   :16918  
##  (Other)       :44                                                        
##              vegetation           location     time        type          
##  Closed           :19   Central Europe:25   Early:20   Length:75         
##  Mixed            :31   Southwest Asia:14   Late :25   Class :character  
##  No info available: 1   Western Europe:36   MIS 2: 7   Mode  :character  
##  Open             :24                       MIS 3:16                     
##                                             MIS 5: 7                     
##                                                                          
##
```

```
table(dat$site,dat$type)
```

```
##                   
##                    EMH Neandertals
##   Amud               0           2
##   Arcy               0           2
##   BD                 0           2
##   Biache             0           1
##   Combe              0           1
##   Kebara             0           1
##   Krapina            0          10
##   Kulna              0           1
##   La Ferrassie       0           2
##   La Quina           0           1
##   Le Moustier        0           1
##   Le Petit           0           1
##   Marillac           0           1
##   Monsempron         0           1
##   Moula              0           3
##   Ochoz              0           1
##   Pontnewydd         0           1
##   Shanidar           0           1
##   St. Cesaire        0           1
##   Suard              0           1
##   Subalyuk           0           1
##   Tabun              0           2
##   Vindija            0           4
##   Zafarraya          0           3
##   Brassempouy        2           0
##   DolniVestonice     4           0
##   Farincourt         1           0
##   GrotteDesEnfants   1           0
##   Isturitz           1           0
##   Lachaud            2           0
##   LesRois            5           0
##   Ohalo              1           0
##   Pavlov             4           0
##   Qafzeh             4           0
##   RoundDuBary        1           0
##   SaintGermain       1           0
##   Skhul              3           0
```

```
table(dat$vegetation,dat$type)
```

```
##                    
##                     EMH Neandertals
##   Closed              0          19
##   Mixed              17          14
##   No info available   0           1
##   Open               13          11
```

```
table(dat$location,dat$type)
```

```
##                 
##                  EMH Neandertals
##   Central Europe   8          17
##   Southwest Asia   8           6
##   Western Europe  14          22
```

```
table(dat$time,dat$type)
```

```
##        
##         EMH Neandertals
##   Early   0          20
##   Late    0          25
##   MIS 2   7           0
##   MIS 3  16           0
##   MIS 5   7           0
```

```
#Make "No info available" an NA.  
dat$vegetation <- as.character(dat$vegetation)
dat$vegetation[dat$vegetation=="No info available"] <- NA
dat$vegetation <- as.factor(dat$vegetation)

dat$type <- as.factor(dat$type)
```

# Table 1

```
#Table 1
library(dplyr)
```

```
## 
## Attaching package: 'dplyr'
```

```
## The following objects are masked from 'package:stats':
## 
##     filter, lag
```

```
## The following objects are masked from 'package:base':
## 
##     intersect, setdiff, setequal, union
```

```
subset(dat, type == "EMH") %>% group_by(site, vegetation, location, time) %>% summarise(count = n())
```

```
## # A tibble: 13 x 5
## # Groups:   site, vegetation, location [?]
##    site             vegetation location       time  count
##    <fct>            <fct>      <fct>          <fct> <int>
##  1 Brassempouy      Open       Western Europe MIS 3     2
##  2 DolniVestonice   Mixed      Central Europe MIS 3     4
##  3 Farincourt       Open       Western Europe MIS 2     1
##  4 GrotteDesEnfants Open       Western Europe MIS 3     1
##  5 Isturitz         Mixed      Western Europe MIS 2     1
##  6 Lachaud          Open       Western Europe MIS 2     2
##  7 LesRois          Open       Western Europe MIS 3     5
##  8 Ohalo            Mixed      Southwest Asia MIS 2     1
##  9 Pavlov           Mixed      Central Europe MIS 3     4
## 10 Qafzeh           Mixed      Southwest Asia MIS 5     4
## 11 RoundDuBary      Open       Western Europe MIS 2     1
## 12 SaintGermain     Open       Western Europe MIS 2     1
## 13 Skhul            Mixed      Southwest Asia MIS 5     3
```

# Table 2

```
#Table 2
library(dplyr)
subset(dat, type == "neandertals") %>% group_by(site, vegetation, location, time) %>% summarise(count = n())
```

```
## # A tibble: 0 x 5
## # Groups:   site, vegetation, location [?]
## # ... with 5 variables: site <fct>, vegetation <fct>, location <fct>,
## #   time <fct>, count <int>
```

```
#Table 4
library(dplyr)
subset(dat, type == "EMH") %>% summarise(mean_epLsar = mean(epLsar), 
                                         median_epLsar = median(epLsar), 
                                         sd_epLsar = sd(epLsar),
                                         mean_Tfv = mean(Tfv), 
                                         median_Tfv = median(Tfv), 
                                         sd_Tfv = sd(Tfv))
```

```
##   mean_epLsar median_epLsar   sd_epLsar mean_Tfv median_Tfv   sd_Tfv
## 1 0.003236333    0.00300875 0.001295136 9520.104   11071.43 4620.413
```

```
subset(dat, type == "neandertals") %>% summarise(mean_epLsar = mean(epLsar), 
                                         median_epLsar = median(epLsar), 
                                         sd_epLsar = sd(epLsar),
                                         mean_Tfv = mean(Tfv), 
                                         median_Tfv = median(Tfv), 
                                         sd_Tfv = sd(Tfv))
```

```
##   mean_epLsar median_epLsar sd_epLsar mean_Tfv median_Tfv sd_Tfv
## 1         NaN            NA        NA      NaN         NA     NA
```

```
#Table 5A
library(dplyr)
library(dplyr)
dat %>% group_by(type, vegetation) %>% summarise(mean_epLsar = mean(epLsar), 
                                         median_epLsar = median(epLsar), 
                                         sd_epLsar = sd(epLsar),
                                         mean_Tfv = mean(Tfv), 
                                         median_Tfv = median(Tfv), 
                                         sd_Tfv = sd(Tfv),
                                         count = n())
```

```
## # A tibble: 6 x 9
## # Groups:   type [?]
##   type  vegetation mean_epLsar median_epLsar sd_epLsar mean_Tfv median_Tfv
##   <fct> <fct>            <dbl>         <dbl>     <dbl>    <dbl>      <dbl>
## 1 EMH   Mixed          0.00307       0.00265  0.00138     9865.     10628.
## 2 EMH   Open           0.00346       0.00354  0.00119     9069.     11515.
## 3 Nean… Closed         0.00356       0.0038   0.00134     8381.      9504.
## 4 Nean… Mixed          0.00306       0.0028   0.00150    10894.     12603.
## 5 Nean… Open           0.00219       0.0021   0.000869   12205.     12423.
## 6 Nean… <NA>           0.0027        0.0027  NA           9296.      9296.
## # ... with 2 more variables: sd_Tfv <dbl>, count <int>
```

```
#Table 5B
dat %>% group_by(type,location) %>% summarise(mean_epLsar = mean(epLsar), 
                                         median_epLsar = median(epLsar), 
                                         sd_epLsar = sd(epLsar),
                                         mean_Tfv = mean(Tfv), 
                                         median_Tfv = median(Tfv), 
                                         sd_Tfv = sd(Tfv),
                                         count = n())
```

```
## # A tibble: 6 x 9
## # Groups:   type [?]
##   type   location  mean_epLsar median_epLsar sd_epLsar mean_Tfv median_Tfv
##   <fct>  <fct>           <dbl>         <dbl>     <dbl>    <dbl>      <dbl>
## 1 EMH    Central …     0.00320       0.00241   0.00174    9278.      8738.
## 2 EMH    Southwes…     0.00293       0.00271   0.00113   10133.     11276.
## 3 EMH    Western …     0.00343       0.00335   0.00115    9308.     11573.
## 4 Neand… Central …     0.00329       0.0031    0.00137    8671.      9661.
## 5 Neand… Southwes…     0.00343       0.00350   0.00139    9424.     12660.
## 6 Neand… Western …     0.00277       0.0025    0.00137   11425.     11749.
## # ... with 2 more variables: sd_Tfv <dbl>, count <int>
```

```
#Table 5C
dat %>% group_by(type,time) %>% summarise(mean_epLsar = mean(epLsar), 
                                         median_epLsar = median(epLsar), 
                                         sd_epLsar = sd(epLsar),
                                         mean_Tfv = mean(Tfv), 
                                         median_Tfv = median(Tfv), 
                                         sd_Tfv = sd(Tfv),
                                         count = n())
```

```
## # A tibble: 5 x 9
## # Groups:   type [?]
##   type       time  mean_epLsar median_epLsar sd_epLsar mean_Tfv median_Tfv
##   <fct>      <fct>       <dbl>         <dbl>     <dbl>    <dbl>      <dbl>
## 1 EMH        MIS 2     0.00325       0.00250   0.00150    9026.     11515.
## 2 EMH        MIS 3     0.00334       0.00331   0.00131    9732.     11104.
## 3 EMH        MIS 5     0.00299       0.00293   0.00121    9530.     10628.
## 4 Neanderta… Early     0.00306       0.0027    0.00106    9099.     10234.
## 5 Neanderta… Late      0.00305       0.0028    0.00160   10933.     12095.
## # ... with 2 more variables: sd_Tfv <dbl>, count <int>
```

```
########################################
##EMH analysis 
########################################
emh <- dat[dat$type=="EMH",]

########################################
##Table 6A
########################################
########################################
##Linear models (One-way ANOVA)
########################################
#Habitat Model
emhVegModel <- lm(epLsar ~ vegetation, data=emh)
summary(emhVegModel)
```

```
## 
## Call:
## lm(formula = epLsar ~ vegetation, data = emh)
## 
## Residuals:
##        Min         1Q     Median         3Q        Max 
## -0.0018017 -0.0010148 -0.0002535  0.0007989  0.0031163 
## 
## Coefficients:
##                 Estimate Std. Error t value Pr(>|t|)    
## (Intercept)    0.0030682  0.0003160   9.709 1.84e-10 ***
## vegetationOpen 0.0003880  0.0004801   0.808    0.426    
## ---
## Signif. codes:  0 '***' 0.001 '**' 0.01 '*' 0.05 '.' 0.1 ' ' 1
## 
## Residual standard error: 0.001303 on 28 degrees of freedom
## Multiple R-squared:  0.0228, Adjusted R-squared:  -0.0121 
## F-statistic: 0.6532 on 1 and 28 DF,  p-value: 0.4258
```

```
#Checking model assumptions
plot(emhVegModel, which = 1)
```

```
plot(emhVegModel, which = 2)
```

```
plot(emhVegModel, which = 3)
```

```
plot(emhVegModel, which = 4)
```

```
#Location Model
emhLocModel <- lm(epLsar ~ location, data=emh)
summary(emhLocModel)
```

```
## 
## Call:
## lm(formula = epLsar ~ location, data = emh)
## 
## Residuals:
##        Min         1Q     Median         3Q        Max 
## -0.0016611 -0.0010011 -0.0003156  0.0008821  0.0029859 
## 
## Coefficients:
##                          Estimate Std. Error t value Pr(>|t|)    
## (Intercept)             0.0031986  0.0004681   6.834 2.43e-07 ***
## locationSouthwest Asia -0.0002711  0.0006619  -0.410    0.685    
## locationWestern Europe  0.0002357  0.0005867   0.402    0.691    
## ---
## Signif. codes:  0 '***' 0.001 '**' 0.01 '*' 0.05 '.' 0.1 ' ' 1
## 
## Residual standard error: 0.001324 on 27 degrees of freedom
## Multiple R-squared:  0.0272, Adjusted R-squared:  -0.04486 
## F-statistic: 0.3774 on 2 and 27 DF,  p-value: 0.6892
```

```
#Checking model assumptions
plot(emhLocModel, which = 1)
```

```
plot(emhLocModel, which = 2)
```

```
plot(emhLocModel, which = 3)
```

```
plot(emhLocModel, which = 4)
```

```
#Time model 
emhTimeModel <- lm(epLsar ~ time, data=emh)
summary(emhTimeModel)
```

```
## 
## Call:
## lm(formula = epLsar ~ time, data = emh)
## 
## Residuals:
##        Min         1Q     Median         3Q        Max 
## -0.0017228 -0.0011171 -0.0001742  0.0009055  0.0028441 
## 
## Coefficients:
##               Estimate Std. Error t value Pr(>|t|)    
## (Intercept)  3.246e-03  5.042e-04   6.437 6.74e-07 ***
## timeMIS 3    9.491e-05  6.045e-04   0.157    0.876    
## timeMIS 5   -2.562e-04  7.130e-04  -0.359    0.722    
## ---
## Signif. codes:  0 '***' 0.001 '**' 0.01 '*' 0.05 '.' 0.1 ' ' 1
## 
## Residual standard error: 0.001334 on 27 degrees of freedom
## Multiple R-squared:  0.01236,    Adjusted R-squared:  -0.0608 
## F-statistic: 0.1689 on 2 and 27 DF,  p-value: 0.8455
```

```
#Checking model assumptions
plot(emhTimeModel, which = 1)
```

```
plot(emhTimeModel, which = 2)
```

```
plot(emhTimeModel, which = 3)
```

```
plot(emhTimeModel, which = 4)
```

```
########################################
##Table 6B
########################################
########################################
##Linear models (One-way ANOVA)
########################################
#Habitat Model
emhVegModel <- lm(Tfv ~ vegetation, data=emh)
summary(emhVegModel)
```

```
## 
## Call:
## lm(formula = Tfv ~ vegetation, data = emh)
## 
## Residuals:
##    Min     1Q Median     3Q    Max 
##  -9865  -4032   1411   3195   7666 
## 
## Coefficients:
##                Estimate Std. Error t value Pr(>|t|)    
## (Intercept)      9864.8     1136.1   8.683 1.97e-09 ***
## vegetationOpen   -795.3     1725.9  -0.461    0.648    
## ---
## Signif. codes:  0 '***' 0.001 '**' 0.01 '*' 0.05 '.' 0.1 ' ' 1
## 
## Residual standard error: 4684 on 28 degrees of freedom
## Multiple R-squared:  0.007527,   Adjusted R-squared:  -0.02792 
## F-statistic: 0.2124 on 1 and 28 DF,  p-value: 0.6485
```

```
#Checking model assumptions
plot(emhVegModel, which = 1)
```

```
plot(emhVegModel, which = 2)
```

```
plot(emhVegModel, which = 3)
```

```
plot(emhVegModel, which = 4)
```

```
#Location Model
emhLocModel <- lm(Tfv ~ location, data=emh)
summary(emhLocModel)
```

```
## 
## Call:
## lm(formula = Tfv ~ location, data = emh)
## 
## Residuals:
##    Min     1Q Median     3Q    Max 
## -10133  -4087   1544   3386   7428 
## 
## Coefficients:
##                        Estimate Std. Error t value Pr(>|t|)    
## (Intercept)             9278.37    1687.37   5.499    8e-06 ***
## locationSouthwest Asia   854.39    2386.30   0.358    0.723    
## locationWestern Europe    29.77    2115.23   0.014    0.989    
## ---
## Signif. codes:  0 '***' 0.001 '**' 0.01 '*' 0.05 '.' 0.1 ' ' 1
## 
## Residual standard error: 4773 on 27 degrees of freedom
## Multiple R-squared:  0.006621,   Adjusted R-squared:  -0.06696 
## F-statistic: 0.08998 on 2 and 27 DF,  p-value: 0.9142
```

```
#Checking model assumptions
plot(emhLocModel, which = 1)
```

```
plot(emhLocModel, which = 2)
```

```
plot(emhLocModel, which = 3)
```

```
plot(emhLocModel, which = 4)
```

```
#Time model 
emhTimeModel <- lm(Tfv ~ time, data=emh)
summary(emhTimeModel)
```

```
## 
## Call:
## lm(formula = Tfv ~ time, data = emh)
## 
## Residuals:
##    Min     1Q Median     3Q    Max 
##  -9530  -3748   1499   3373   7004 
## 
## Coefficients:
##             Estimate Std. Error t value Pr(>|t|)    
## (Intercept)   9026.2     1806.3   4.997 3.07e-05 ***
## timeMIS 3      705.9     2165.7   0.326    0.747    
## timeMIS 5      503.3     2554.5   0.197    0.845    
## ---
## Signif. codes:  0 '***' 0.001 '**' 0.01 '*' 0.05 '.' 0.1 ' ' 1
## 
## Residual standard error: 4779 on 27 degrees of freedom
## Multiple R-squared:  0.00392,    Adjusted R-squared:  -0.06986 
## F-statistic: 0.05313 on 2 and 27 DF,  p-value: 0.9484
```

```
#Checking model assumptions
plot(emhTimeModel, which = 1)
```

```
plot(emhTimeModel, which = 2)
```

```
plot(emhTimeModel, which = 3)
```

```
plot(emhTimeModel, which = 4)
```

# Table 7

```
########################################
##epLsar: Linear models (One-way ANOVA)
########################################
typeModel<-lm(epLsar ~ type,data=dat)
summary(typeModel)
```

```
## 
## Call:
## lm(formula = epLsar ~ type, data = dat)
## 
## Residuals:
##        Min         1Q     Median         3Q        Max 
## -0.0025533 -0.0010703 -0.0003063  0.0008467  0.0037467 
## 
## Coefficients:
##                   Estimate Std. Error t value Pr(>|t|)    
## (Intercept)      0.0032363  0.0002448  13.219   <2e-16 ***
## typeNeandertals -0.0001830  0.0003161  -0.579    0.564    
## ---
## Signif. codes:  0 '***' 0.001 '**' 0.01 '*' 0.05 '.' 0.1 ' ' 1
## 
## Residual standard error: 0.001341 on 73 degrees of freedom
## Multiple R-squared:  0.004571,   Adjusted R-squared:  -0.009065 
## F-statistic: 0.3352 on 1 and 73 DF,  p-value: 0.5644
```

```
#Check assumptions
plot(typeModel,which=1)
```

```
plot(typeModel,which=2)
```

```
plot(typeModel,which=3)
```

```
plot(typeModel,which=4)
```

```
########################################
##Tfv: Linear models (One-way ANOVA)
########################################
typeModel <- lm(Tfv ~ type,data=dat)
summary(typeModel)
```

```
## 
## Call:
## lm(formula = Tfv ~ type, data = dat)
## 
## Residuals:
##    Min     1Q Median     3Q    Max 
## -10118  -3537   1108   3151   7216 
## 
## Coefficients:
##                 Estimate Std. Error t value Pr(>|t|)    
## (Intercept)       9520.1      813.8  11.698   <2e-16 ***
## typeNeandertals    597.7     1050.6   0.569    0.571    
## ---
## Signif. codes:  0 '***' 0.001 '**' 0.01 '*' 0.05 '.' 0.1 ' ' 1
## 
## Residual standard error: 4457 on 73 degrees of freedom
## Multiple R-squared:  0.004413,   Adjusted R-squared:  -0.009225 
## F-statistic: 0.3236 on 1 and 73 DF,  p-value: 0.5712
```

```
#Check assumptions
plot(typeModel,which=1)
```

```
plot(typeModel,which=2)
```

```
plot(typeModel,which=3)
```

```
plot(typeModel,which=4)
```

# Table 8

```
#Table 8A
########################################
##Two-way ANOVA (Dropping Vegetation == Closed)
########################################
allModel_epLsar_noclosed <- lm(epLsar ~ vegetation*type, data=subset(dat, vegetation != "Closed"))
summary(allModel_epLsar_noclosed)
```

```
## 
## Call:
## lm(formula = epLsar ~ vegetation * type, data = subset(dat, vegetation != 
##     "Closed"))
## 
## Residuals:
##        Min         1Q     Median         3Q        Max 
## -0.0018017 -0.0008752 -0.0002643  0.0006748  0.0037357 
## 
## Coefficients:
##                                  Estimate Std. Error t value Pr(>|t|)    
## (Intercept)                     3.068e-03  3.120e-04   9.835 2.27e-13 ***
## vegetationOpen                  3.880e-04  4.739e-04   0.819   0.4168    
## typeNeandertals                -3.920e-06  4.642e-04  -0.008   0.9933    
## vegetationOpen:typeNeandertals -1.261e-03  7.023e-04  -1.796   0.0784 .  
## ---
## Signif. codes:  0 '***' 0.001 '**' 0.01 '*' 0.05 '.' 0.1 ' ' 1
## 
## Residual standard error: 0.001286 on 51 degrees of freedom
## Multiple R-squared:  0.1062, Adjusted R-squared:  0.05365 
## F-statistic:  2.02 on 3 and 51 DF,  p-value: 0.1227
```

```
#Check assumptions
plot(allModel_epLsar_noclosed,which=1)
```

```
plot(allModel_epLsar_noclosed,which=2)
```

```
plot(allModel_epLsar_noclosed,which=3)
```

```
plot(allModel_epLsar_noclosed,which=4)
```

```
#Table 8B
library(MASS)
```

```
## 
## Attaching package: 'MASS'
```

```
## The following object is masked from 'package:dplyr':
## 
##     select
```

```
dat$vegetation <- as.character(dat$vegetation)
rrHuber <- rlm(epLsar ~ vegetation*type, data =dat[!is.na(dat$vegetation) & dat$vegetation != "Closed",])
summary(rrHuber)
```

```
## 
## Call: rlm(formula = epLsar ~ vegetation * type, data = dat[!is.na(dat$vegetation) & 
##     dat$vegetation != "Closed", ])
## Residuals:
##        Min         1Q     Median         3Q        Max 
## -1.700e-03 -7.661e-04 -3.268e-05  7.571e-04  3.967e-03 
## 
## Coefficients:
##                                Value   Std. Error t value
## (Intercept)                     0.0029  0.0003     9.6727
## vegetationOpen                  0.0005  0.0005     1.0676
## typeNeandertals                -0.0001  0.0004    -0.1811
## vegetationOpen:typeNeandertals -0.0011  0.0007    -1.6529
## 
## Residual standard error: 0.001187 on 51 degrees of freedom
```

```
2*(1-pnorm(abs(summary(rrHuber)$coefficient[,3])))
```

```
##                    (Intercept)                 vegetationOpen 
##                     0.00000000                     0.28567907 
##                typeNeandertals vegetationOpen:typeNeandertals 
##                     0.85625438                     0.09835957
```

```
#Final Huber Weights
hweights <- data.frame(dat[!is.na(dat$vegetation) & dat$vegetation != "Closed",], resid = rrHuber$resid, weight = rrHuber$w)
hweights2 <- hweights[order(rrHuber$w), ]
hweights2
```

```
##                site   specimen    epLsar       Tfv vegetation
## 14          Vindija       12.7 0.0068000 12094.760      Mixed
## 66           Pavlov    5slash1 0.0061845 15591.576      Mixed
## 50   DolniVestonice         15 0.0054975  6034.072      Mixed
## 40         Shanidar          3 0.0053000  1172.640      Mixed
## 55          Lachaud          4 0.0055190  2165.205       Open
## 56          Lachaud          5 0.0051765  6438.888       Open
## 27      Le Moustier          1 0.0005000 10308.820       Open
## 74            Skhul          4 0.0012665  5765.140      Mixed
## 70           Qafzeh          9 0.0045230 10628.356      Mixed
## 11          Vindija       12.2 0.0025000 10952.870      Mixed
## 12          Vindija       12.3 0.0034000  9812.160      Mixed
## 13          Vindija       12.4 0.0028000 13689.340      Mixed
## 15            Kulna          1 0.0013000 13077.600      Mixed
## 16            Ochoz          1 0.0028000 14563.420      Mixed
## 17             Arcy          0 0.0018000 14132.420       Open
## 18             Arcy          0 0.0014000 15885.660       Open
## 20            Combe          5 0.0036000 12423.390       Open
## 21            Suard          0 0.0025000 14196.790       Open
## 22               BD         10 0.0027000 11897.110       Open
## 23               BD         12 0.0025000 12113.070       Open
## 24     La Ferrassie          1 0.0026000 16331.090      Mixed
## 25     La Ferrassie          2 0.0032000 12166.670      Mixed
## 26         La Quina          5 0.0018000  6530.290       Open
## 28         Le Petit          3 0.0019000  8611.000       Open
## 29         Marillac          0 0.0033000 14001.810       Open
## 34      St. Cesaire          0 0.0017000  5461.790      Mixed
## 35       Pontnewydd         10 0.0022000 13234.760      Mixed
## 36         Subalyuk          1 0.0021000 14151.770       Open
## 41             Amud          1 0.0013000 13039.350      Mixed
## 42             Amud          2 0.0039000 16918.340      Mixed
## 43           Kebara          2 0.0031000     0.000      Mixed
## 46      Brassempouy       BR90 0.0040485 12077.535       Open
## 47      Brassempouy       BR94 0.0027275  2061.425       Open
## 48   DolniVestonice         13 0.0018490 12632.026      Mixed
## 49   DolniVestonice         14 0.0035615  4848.976      Mixed
## 51   DolniVestonice          3 0.0016275 10577.267      Mixed
## 52       Farincourt          1 0.0020420  3556.818       Open
## 53 GrotteDesEnfants       GdE4 0.0043505  4151.129       Open
## 54         Isturitz        115 0.0031500 12411.731      Mixed
## 57          LesRois          3 0.0030875 16735.674       Open
## 58          LesRois          5 0.0042690 11631.483       Open
## 59          LesRois          7 0.0021500 14808.913       Open
## 60          LesRois   NoNumber 0.0036835 12704.369       Open
## 61          LesRois   NoNumber 0.0035410  7315.665       Open
## 62            Ohalo          2 0.0024955 14355.555      Mixed
## 63           Pavlov          1 0.0021820 13068.702      Mixed
## 64           Pavlov         23 0.0026465  6899.535      Mixed
## 65           Pavlov         25 0.0020405  4574.831      Mixed
## 67           Qafzeh          5 0.0038255 10052.726      Mixed
## 68           Qafzeh          6 0.0029300 11922.771      Mixed
## 69           Qafzeh          7 0.0041015     0.000      Mixed
## 71      RoundDuBary         RB 0.0024800 12740.693       Open
## 72     SaintGermain 1970-07-12 0.0018555 11514.502       Open
## 73            Skhul          2 0.0022825 13033.947      Mixed
## 75            Skhul          5 0.0019960 15303.622      Mixed
##          location  time        type         resid    weight
## 14 Central Europe  Late Neandertals  3.967324e-03 0.4023003
## 66 Central Europe MIS 3         EMH  3.270622e-03 0.4880112
## 50 Central Europe MIS 3         EMH  2.583622e-03 0.6177813
## 40 Southwest Asia  Late Neandertals  2.467324e-03 0.6468785
## 55 Western Europe MIS 2         EMH  2.116535e-03 0.7540896
## 56 Western Europe MIS 2         EMH  1.774035e-03 0.8996769
## 27 Western Europe  Late Neandertals -1.700395e-03 0.9386330
## 74 Southwest Asia MIS 5         EMH -1.647378e-03 0.9687824
## 70 Southwest Asia MIS 5         EMH  1.609122e-03 0.9919395
## 11 Central Europe  Late Neandertals -3.326762e-04 1.0000000
## 12 Central Europe  Late Neandertals  5.673238e-04 1.0000000
## 13 Central Europe  Late Neandertals -3.267617e-05 1.0000000
## 15 Central Europe  Late Neandertals -1.532676e-03 1.0000000
## 16 Central Europe  Late Neandertals -3.267617e-05 1.0000000
## 17 Western Europe  Late Neandertals -4.003953e-04 1.0000000
## 18 Western Europe  Late Neandertals -8.003953e-04 1.0000000
## 20 Western Europe  Late Neandertals  1.399605e-03 1.0000000
## 21 Western Europe Early Neandertals  2.996047e-04 1.0000000
## 22 Western Europe Early Neandertals  4.996047e-04 1.0000000
## 23 Western Europe Early Neandertals  2.996047e-04 1.0000000
## 24 Western Europe  Late Neandertals -2.326762e-04 1.0000000
## 25 Western Europe  Late Neandertals  3.673238e-04 1.0000000
## 26 Western Europe  Late Neandertals -4.003953e-04 1.0000000
## 28 Western Europe  Late Neandertals -3.003953e-04 1.0000000
## 29 Western Europe  Late Neandertals  1.099605e-03 1.0000000
## 34 Western Europe  Late Neandertals -1.132676e-03 1.0000000
## 35 Western Europe Early Neandertals -6.326762e-04 1.0000000
## 36 Central Europe  Late Neandertals -1.003953e-04 1.0000000
## 41 Southwest Asia  Late Neandertals -1.532676e-03 1.0000000
## 42 Southwest Asia  Late Neandertals  1.067324e-03 1.0000000
## 43 Southwest Asia  Late Neandertals  2.673238e-04 1.0000000
## 46 Western Europe MIS 3         EMH  6.460350e-04 1.0000000
## 47 Western Europe MIS 3         EMH -6.749650e-04 1.0000000
## 48 Central Europe MIS 3         EMH -1.064878e-03 1.0000000
## 49 Central Europe MIS 3         EMH  6.476220e-04 1.0000000
## 51 Central Europe MIS 3         EMH -1.286378e-03 1.0000000
## 52 Western Europe MIS 2         EMH -1.360465e-03 1.0000000
## 53 Western Europe MIS 3         EMH  9.480350e-04 1.0000000
## 54 Western Europe MIS 2         EMH  2.361220e-04 1.0000000
## 57 Western Europe MIS 3         EMH -3.149650e-04 1.0000000
## 58 Western Europe MIS 3         EMH  8.665350e-04 1.0000000
## 59 Western Europe MIS 3         EMH -1.252465e-03 1.0000000
## 60 Western Europe MIS 3         EMH  2.810350e-04 1.0000000
## 61 Western Europe MIS 3         EMH  1.385350e-04 1.0000000
## 62 Southwest Asia MIS 2         EMH -4.183780e-04 1.0000000
## 63 Central Europe MIS 3         EMH -7.318780e-04 1.0000000
## 64 Central Europe MIS 3         EMH -2.673780e-04 1.0000000
## 65 Central Europe MIS 3         EMH -8.733780e-04 1.0000000
## 67 Southwest Asia MIS 5         EMH  9.116220e-04 1.0000000
## 68 Southwest Asia MIS 5         EMH  1.612198e-05 1.0000000
## 69 Southwest Asia MIS 5         EMH  1.187622e-03 1.0000000
## 71 Western Europe MIS 2         EMH -9.224650e-04 1.0000000
## 72 Western Europe MIS 2         EMH -1.546965e-03 1.0000000
## 73 Southwest Asia MIS 5         EMH -6.313780e-04 1.0000000
## 75 Southwest Asia MIS 5         EMH -9.178780e-04 1.0000000
```

```
#Table 8C
preds <- data.frame(type = c(rep("Neandertals",2),rep("EMH",2)), vegetation = c("Mixed","Open","Mixed","Open"))
preds$yhat <- predict(allModel_epLsar_noclosed,newdata=preds, interval = "confidence")
preds
```

```
##          type vegetation    yhat.fit    yhat.lwr    yhat.upr
## 1 Neandertals      Mixed 0.003064286 0.002374150 0.003754422
## 2 Neandertals       Open 0.002190909 0.001412331 0.002969487
## 3         EMH      Mixed 0.003068206 0.002441918 0.003694494
## 4         EMH       Open 0.003456192 0.002740004 0.004172380
```

# Table 9

```
#Table 9A
########################################
##Two-way ANOVA (Dropping Vegetation == Closed)
########################################
allModel_Tfv_noclosed <- lm(Tfv ~ vegetation*type, data=subset(dat, vegetation != "Closed"))
summary(allModel_Tfv_noclosed)
```

```
## 
## Call:
## lm(formula = Tfv ~ vegetation * type, data = subset(dat, vegetation != 
##     "Closed"))
## 
## Residuals:
##    Min     1Q Median     3Q    Max 
## -10894  -3280   1273   2902   7666 
## 
## Coefficients:
##                                Estimate Std. Error t value Pr(>|t|)    
## (Intercept)                      9864.8     1095.5   9.005 4.05e-12 ***
## vegetationOpen                   -795.3     1664.2  -0.478    0.635    
## typeNeandertals                  1029.2     1630.1   0.631    0.531    
## vegetationOpen:typeNeandertals   2106.2     2466.0   0.854    0.397    
## ---
## Signif. codes:  0 '***' 0.001 '**' 0.01 '*' 0.05 '.' 0.1 ' ' 1
## 
## Residual standard error: 4517 on 51 degrees of freedom
## Multiple R-squared:  0.06061,    Adjusted R-squared:  0.005348 
## F-statistic: 1.097 on 3 and 51 DF,  p-value: 0.359
```

```
#Check assumptions
plot(allModel_Tfv_noclosed,which=1)
```

```
plot(allModel_Tfv_noclosed,which=2)
```

```
plot(allModel_Tfv_noclosed,which=3)
```

```
plot(allModel_Tfv_noclosed,which=4)
```

```
#Table 9B
library(MASS)
dat$vegetation <- as.character(dat$vegetation)
rrHuber <- rlm(Tfv ~ vegetation*type, data =dat[!is.na(dat$vegetation) & dat$vegetation != "Closed",])
summary(rrHuber)
```

```
## 
## Call: rlm(formula = Tfv ~ vegetation * type, data = dat[!is.na(dat$vegetation) & 
##     dat$vegetation != "Closed", ])
## Residuals:
##      Min       1Q   Median       3Q      Max 
## -11675.9  -3406.0    510.5   2703.3   7623.2 
## 
## Coefficients:
##                                Value      Std. Error t value   
## (Intercept)                    10117.8652  1072.2711     9.4359
## vegetationOpen                 -1005.4248  1628.8967    -0.6172
## typeNeandertals                 1557.9881  1595.5904     0.9764
## vegetationOpen:typeNeandertals  1534.3107  2413.7850     0.6356
## 
## Residual standard error: 4323 on 51 degrees of freedom
```

```
2*(1-pnorm(abs(summary(rrHuber)$coefficient[,3])))
```

```
##                    (Intercept)                 vegetationOpen 
##                      0.0000000                      0.5370746 
##                typeNeandertals vegetationOpen:typeNeandertals 
##                      0.3288496                      0.5250077
```

```
#Final Huber Weights
hweights <- data.frame(dat[!is.na(dat$vegetation) & dat$vegetation != "Closed",], resid = rrHuber$resid, weight = rrHuber$w)
hweights2 <- hweights[order(rrHuber$w), ]
hweights2
```

```
##                site   specimen    epLsar       Tfv vegetation
## 43           Kebara          2 0.0031000     0.000      Mixed
## 40         Shanidar          3 0.0053000  1172.640      Mixed
## 69           Qafzeh          7 0.0041015     0.000      Mixed
## 57          LesRois          3 0.0030875 16735.674       Open
## 47      Brassempouy       BR94 0.0027275  2061.425       Open
## 55          Lachaud          4 0.0055190  2165.205       Open
## 34      St. Cesaire          0 0.0017000  5461.790      Mixed
## 11          Vindija       12.2 0.0025000 10952.870      Mixed
## 12          Vindija       12.3 0.0034000  9812.160      Mixed
## 13          Vindija       12.4 0.0028000 13689.340      Mixed
## 14          Vindija       12.7 0.0068000 12094.760      Mixed
## 15            Kulna          1 0.0013000 13077.600      Mixed
## 16            Ochoz          1 0.0028000 14563.420      Mixed
## 17             Arcy          0 0.0018000 14132.420       Open
## 18             Arcy          0 0.0014000 15885.660       Open
## 20            Combe          5 0.0036000 12423.390       Open
## 21            Suard          0 0.0025000 14196.790       Open
## 22               BD         10 0.0027000 11897.110       Open
## 23               BD         12 0.0025000 12113.070       Open
## 24     La Ferrassie          1 0.0026000 16331.090      Mixed
## 25     La Ferrassie          2 0.0032000 12166.670      Mixed
## 26         La Quina          5 0.0018000  6530.290       Open
## 27      Le Moustier          1 0.0005000 10308.820       Open
## 28         Le Petit          3 0.0019000  8611.000       Open
## 29         Marillac          0 0.0033000 14001.810       Open
## 35       Pontnewydd         10 0.0022000 13234.760      Mixed
## 36         Subalyuk          1 0.0021000 14151.770       Open
## 41             Amud          1 0.0013000 13039.350      Mixed
## 42             Amud          2 0.0039000 16918.340      Mixed
## 46      Brassempouy       BR90 0.0040485 12077.535       Open
## 48   DolniVestonice         13 0.0018490 12632.026      Mixed
## 49   DolniVestonice         14 0.0035615  4848.976      Mixed
## 50   DolniVestonice         15 0.0054975  6034.072      Mixed
## 51   DolniVestonice          3 0.0016275 10577.267      Mixed
## 52       Farincourt          1 0.0020420  3556.818       Open
## 53 GrotteDesEnfants       GdE4 0.0043505  4151.129       Open
## 54         Isturitz        115 0.0031500 12411.731      Mixed
## 56          Lachaud          5 0.0051765  6438.888       Open
## 58          LesRois          5 0.0042690 11631.483       Open
## 59          LesRois          7 0.0021500 14808.913       Open
## 60          LesRois   NoNumber 0.0036835 12704.369       Open
## 61          LesRois   NoNumber 0.0035410  7315.665       Open
## 62            Ohalo          2 0.0024955 14355.555      Mixed
## 63           Pavlov          1 0.0021820 13068.702      Mixed
## 64           Pavlov         23 0.0026465  6899.535      Mixed
## 65           Pavlov         25 0.0020405  4574.831      Mixed
## 66           Pavlov    5slash1 0.0061845 15591.576      Mixed
## 67           Qafzeh          5 0.0038255 10052.726      Mixed
## 68           Qafzeh          6 0.0029300 11922.771      Mixed
## 70           Qafzeh          9 0.0045230 10628.356      Mixed
## 71      RoundDuBary         RB 0.0024800 12740.693       Open
## 72     SaintGermain 1970-07-12 0.0018555 11514.502       Open
## 73            Skhul          2 0.0022825 13033.947      Mixed
## 74            Skhul          4 0.0012665  5765.140      Mixed
## 75            Skhul          5 0.0019960 15303.622      Mixed
##          location  time        type        resid    weight
## 43 Southwest Asia  Late Neandertals -11675.85327 0.4980565
## 40 Southwest Asia  Late Neandertals -10503.21327 0.5536653
## 69 Southwest Asia MIS 5         EMH -10117.86519 0.5747250
## 57 Western Europe MIS 3         EMH   7623.23394 0.7627688
## 47 Western Europe MIS 3         EMH  -7051.01534 0.8247290
## 55 Western Europe MIS 2         EMH  -6947.23576 0.8370495
## 34 Western Europe  Late Neandertals  -6214.06327 0.9358569
## 11 Central Europe  Late Neandertals   -722.98327 1.0000000
## 12 Central Europe  Late Neandertals  -1863.69327 1.0000000
## 13 Central Europe  Late Neandertals   2013.48673 1.0000000
## 14 Central Europe  Late Neandertals    418.90673 1.0000000
## 15 Central Europe  Late Neandertals   1401.74673 1.0000000
## 16 Central Europe  Late Neandertals   2887.56673 1.0000000
## 17 Western Europe  Late Neandertals   1927.68091 1.0000000
## 18 Western Europe  Late Neandertals   3680.92091 1.0000000
## 20 Western Europe  Late Neandertals    218.65091 1.0000000
## 21 Western Europe Early Neandertals   1992.05091 1.0000000
## 22 Western Europe Early Neandertals   -307.62909 1.0000000
## 23 Western Europe Early Neandertals    -91.66909 1.0000000
## 24 Western Europe  Late Neandertals   4655.23673 1.0000000
## 25 Western Europe  Late Neandertals    490.81673 1.0000000
## 26 Western Europe  Late Neandertals  -5674.44909 1.0000000
## 27 Western Europe  Late Neandertals  -1895.91909 1.0000000
## 28 Western Europe  Late Neandertals  -3593.73909 1.0000000
## 29 Western Europe  Late Neandertals   1797.07091 1.0000000
## 35 Western Europe Early Neandertals   1558.90673 1.0000000
## 36 Central Europe  Late Neandertals   1947.03091 1.0000000
## 41 Southwest Asia  Late Neandertals   1363.49673 1.0000000
## 42 Southwest Asia  Late Neandertals   5242.48673 1.0000000
## 46 Western Europe MIS 3         EMH   2965.09495 1.0000000
## 48 Central Europe MIS 3         EMH   2514.16053 1.0000000
## 49 Central Europe MIS 3         EMH  -5268.88926 1.0000000
## 50 Central Europe MIS 3         EMH  -4083.79337 1.0000000
## 51 Central Europe MIS 3         EMH    459.40197 1.0000000
## 52 Western Europe MIS 2         EMH  -5555.62190 1.0000000
## 53 Western Europe MIS 3         EMH  -4961.31155 1.0000000
## 54 Western Europe MIS 2         EMH   2293.86590 1.0000000
## 56 Western Europe MIS 2         EMH  -2673.55280 1.0000000
## 58 Western Europe MIS 3         EMH   2519.04267 1.0000000
## 59 Western Europe MIS 3         EMH   5696.47298 1.0000000
## 60 Western Europe MIS 3         EMH   3591.92896 1.0000000
## 61 Western Europe MIS 3         EMH  -1796.77553 1.0000000
## 62 Southwest Asia MIS 2         EMH   4237.68985 1.0000000
## 63 Central Europe MIS 3         EMH   2950.83722 1.0000000
## 64 Central Europe MIS 3         EMH  -3218.33055 1.0000000
## 65 Central Europe MIS 3         EMH  -5543.03395 1.0000000
## 66 Central Europe MIS 3         EMH   5473.71129 1.0000000
## 67 Southwest Asia MIS 5         EMH    -65.13960 1.0000000
## 68 Southwest Asia MIS 5         EMH   1804.90625 1.0000000
## 70 Southwest Asia MIS 5         EMH    510.49064 1.0000000
## 71 Western Europe MIS 2         EMH   3628.25260 1.0000000
## 72 Western Europe MIS 2         EMH   2402.06138 1.0000000
## 73 Southwest Asia MIS 5         EMH   2916.08156 1.0000000
## 74 Southwest Asia MIS 5         EMH  -4352.72532 1.0000000
## 75 Southwest Asia MIS 5         EMH   5185.75651 1.0000000
```

```
#Table 9C
preds <- data.frame(type = c(rep("Neandertals",2),rep("EMH",2)), vegetation = c("Mixed","Open","Mixed","Open"))
preds$yhat <- predict(allModel_Tfv_noclosed,newdata=preds, interval = "confidence")
preds
```

```
##          type vegetation  yhat.fit  yhat.lwr  yhat.upr
## 1 Neandertals      Mixed 10893.914  8470.442 13317.385
## 2 Neandertals       Open 12204.739  9470.695 14938.784
## 3         EMH      Mixed  9864.755  7665.490 12064.019
## 4         EMH       Open  9069.408  6554.452 11584.363
```

# Table 10

```
#Table 10A
########################################
##Two-way ANOVA 
########################################
allModel_epLsar <- lm(epLsar ~ location*type, data=dat)
summary(allModel_epLsar)
```

```
## 
## Call:
## lm(formula = epLsar ~ location * type, data = dat)
## 
## Residuals:
##        Min         1Q     Median         3Q        Max 
## -0.0022682 -0.0009613 -0.0002682  0.0008332  0.0035118 
## 
## Coefficients:
##                                          Estimate Std. Error t value
## (Intercept)                             3.199e-03  4.785e-04   6.685
## locationSouthwest Asia                 -2.711e-04  6.766e-04  -0.401
## locationWestern Europe                  2.357e-04  5.998e-04   0.393
## typeNeandertals                         8.961e-05  5.802e-04   0.154
## locationSouthwest Asia:typeNeandertals  4.162e-04  9.331e-04   0.446
## locationWestern Europe:typeNeandertals -7.558e-04  7.421e-04  -1.018
##                                        Pr(>|t|)    
## (Intercept)                            4.92e-09 ***
## locationSouthwest Asia                    0.690    
## locationWestern Europe                    0.696    
## typeNeandertals                           0.878    
## locationSouthwest Asia:typeNeandertals    0.657    
## locationWestern Europe:typeNeandertals    0.312    
## ---
## Signif. codes:  0 '***' 0.001 '**' 0.01 '*' 0.05 '.' 0.1 ' ' 1
## 
## Residual standard error: 0.001353 on 69 degrees of freedom
## Multiple R-squared:  0.04185,    Adjusted R-squared:  -0.02758 
## F-statistic: 0.6027 on 5 and 69 DF,  p-value: 0.698
```

```
#Check assumptions
plot(allModel_epLsar,which=1)
```

```
plot(allModel_epLsar,which=2)
```

```
plot(allModel_epLsar,which=3)
```

```
plot(allModel_epLsar,which=4)
```

```
#Table 10B
library(MASS)
dat$vegetation <- as.character(dat$vegetation)
rrHuber <- rlm(epLsar ~ location*type, data =dat[!is.na(dat$vegetation) ,])
summary(rrHuber)
```

```
## 
## Call: rlm(formula = epLsar ~ location * type, data = dat[!is.na(dat$vegetation), 
##     ])
## Residuals:
##        Min         1Q     Median         3Q        Max 
## -0.0022000 -0.0008199 -0.0001688  0.0008603  0.0036258 
## 
## Coefficients:
##                                        Value   Std. Error t value
## (Intercept)                             0.0029  0.0005     5.8289
## locationSouthwest Asia                  0.0001  0.0007     0.0956
## locationWestern Europe                  0.0005  0.0006     0.8513
## typeNeandertals                         0.0003  0.0006     0.5202
## locationSouthwest Asia:typeNeandertals  0.0003  0.0010     0.2706
## locationWestern Europe:typeNeandertals -0.0011  0.0008    -1.4104
## 
## Residual standard error: 0.001219 on 68 degrees of freedom
```

```
2*(1-pnorm(abs(summary(rrHuber)$coefficient[,3])))
```

```
##                            (Intercept) 
##                           5.578087e-09 
##                 locationSouthwest Asia 
##                           9.238016e-01 
##                 locationWestern Europe 
##                           3.946039e-01 
##                        typeNeandertals 
##                           6.029024e-01 
## locationSouthwest Asia:typeNeandertals 
##                           7.867205e-01 
## locationWestern Europe:typeNeandertals 
##                           1.584323e-01
```

```
#Final Huber Weights
hweights <- data.frame(dat[!is.na(dat$vegetation),], resid = rrHuber$resid, weight = rrHuber$w)
hweights2 <- hweights[order(rrHuber$w), ]
hweights2
```

```
##                site   specimen    epLsar       Tfv vegetation
## 14          Vindija       12.7 0.0068000 12094.760      Mixed
## 66           Pavlov    5slash1 0.0061845 15591.576      Mixed
## 39        Zafarraya          0 0.0056000  6304.950     Closed
## 37        Zafarraya          0 0.0054000 11348.030     Closed
## 38        Zafarraya          0 0.0054000 11041.150     Closed
## 50   DolniVestonice         15 0.0054975  6034.072      Mixed
## 41             Amud          1 0.0013000 13039.350      Mixed
## 55          Lachaud          4 0.0055190  2165.205       Open
## 27      Le Moustier          1 0.0005000 10308.820       Open
## 15            Kulna          1 0.0013000 13077.600      Mixed
## 10          Krapina        132 0.0050000 11031.710     Closed
## 40         Shanidar          3 0.0053000  1172.640      Mixed
## 56          Lachaud          5 0.0051765  6438.888       Open
## 33            Moula          0 0.0043000 10806.620     Closed
## 74            Skhul          4 0.0012665  5765.140      Mixed
## 1           Krapina        131 0.0017000  4261.490     Closed
## 2           Krapina         49 0.0025000  1039.300     Closed
## 3           Krapina         73 0.0019000  8031.830     Closed
## 4           Krapina         75 0.0038000  9661.460     Closed
## 5           Krapina         55 0.0045000  1185.730     Closed
## 6           Krapina        157 0.0043000  4906.220     Closed
## 7           Krapina         70 0.0031000  5192.490     Closed
## 8           Krapina         92 0.0035000  4255.480     Closed
## 9           Krapina        130 0.0039000  9504.070     Closed
## 11          Vindija       12.2 0.0025000 10952.870      Mixed
## 12          Vindija       12.3 0.0034000  9812.160      Mixed
## 13          Vindija       12.4 0.0028000 13689.340      Mixed
## 16            Ochoz          1 0.0028000 14563.420      Mixed
## 17             Arcy          0 0.0018000 14132.420       Open
## 18             Arcy          0 0.0014000 15885.660       Open
## 19           Biache          1 0.0023000  8584.140     Closed
## 20            Combe          5 0.0036000 12423.390       Open
## 21            Suard          0 0.0025000 14196.790       Open
## 22               BD         10 0.0027000 11897.110       Open
## 23               BD         12 0.0025000 12113.070       Open
## 24     La Ferrassie          1 0.0026000 16331.090      Mixed
## 25     La Ferrassie          2 0.0032000 12166.670      Mixed
## 26         La Quina          5 0.0018000  6530.290       Open
## 28         Le Petit          3 0.0019000  8611.000       Open
## 29         Marillac          0 0.0033000 14001.810       Open
## 31            Moula        259 0.0022000 11600.830     Closed
## 32            Moula        588 0.0013000 15069.990     Closed
## 34      St. Cesaire          0 0.0017000  5461.790      Mixed
## 35       Pontnewydd         10 0.0022000 13234.760      Mixed
## 36         Subalyuk          1 0.0021000 14151.770       Open
## 42             Amud          2 0.0039000 16918.340      Mixed
## 43           Kebara          2 0.0031000     0.000      Mixed
## 44            Tabun          0 0.0027000 13129.820     Closed
## 45            Tabun          0 0.0043000 12281.240     Closed
## 46      Brassempouy       BR90 0.0040485 12077.535       Open
## 47      Brassempouy       BR94 0.0027275  2061.425       Open
## 48   DolniVestonice         13 0.0018490 12632.026      Mixed
## 49   DolniVestonice         14 0.0035615  4848.976      Mixed
## 51   DolniVestonice          3 0.0016275 10577.267      Mixed
## 52       Farincourt          1 0.0020420  3556.818       Open
## 53 GrotteDesEnfants       GdE4 0.0043505  4151.129       Open
## 54         Isturitz        115 0.0031500 12411.731      Mixed
## 57          LesRois          3 0.0030875 16735.674       Open
## 58          LesRois          5 0.0042690 11631.483       Open
## 59          LesRois          7 0.0021500 14808.913       Open
## 60          LesRois   NoNumber 0.0036835 12704.369       Open
## 61          LesRois   NoNumber 0.0035410  7315.665       Open
## 62            Ohalo          2 0.0024955 14355.555      Mixed
## 63           Pavlov          1 0.0021820 13068.702      Mixed
## 64           Pavlov         23 0.0026465  6899.535      Mixed
## 65           Pavlov         25 0.0020405  4574.831      Mixed
## 67           Qafzeh          5 0.0038255 10052.726      Mixed
## 68           Qafzeh          6 0.0029300 11922.771      Mixed
## 69           Qafzeh          7 0.0041015     0.000      Mixed
## 70           Qafzeh          9 0.0045230 10628.356      Mixed
## 71      RoundDuBary         RB 0.0024800 12740.693       Open
## 72     SaintGermain 1970-07-12 0.0018555 11514.502       Open
## 73            Skhul          2 0.0022825 13033.947      Mixed
## 75            Skhul          5 0.0019960 15303.622      Mixed
##          location  time        type         resid    weight
## 14 Central Europe  Late Neandertals  3.625774e-03 0.4520628
## 66 Central Europe MIS 3         EMH  3.320273e-03 0.4936844
## 39 Western Europe  Late Neandertals  2.980149e-03 0.5500152
## 37 Western Europe  Late Neandertals  2.780149e-03 0.5895844
## 38 Western Europe  Late Neandertals  2.780149e-03 0.5895844
## 50 Central Europe MIS 3         EMH  2.633273e-03 0.6224936
## 41 Southwest Asia  Late Neandertals -2.200000e-03 0.7450257
## 55 Western Europe MIS 2         EMH  2.130392e-03 0.7694075
## 27 Western Europe  Late Neandertals -2.119851e-03 0.7731471
## 15 Central Europe  Late Neandertals -1.874226e-03 0.8745026
## 10 Central Europe Early Neandertals  1.825774e-03 0.8977553
## 40 Southwest Asia  Late Neandertals  1.800000e-03 0.9105867
## 56 Western Europe MIS 2         EMH  1.787892e-03 0.9168090
## 33 Western Europe Early Neandertals  1.680149e-03 0.9756172
## 74 Southwest Asia MIS 5         EMH -1.664193e-03 0.9849503
## 1  Central Europe Early Neandertals -1.474226e-03 1.0000000
## 2  Central Europe Early Neandertals -6.742258e-04 1.0000000
## 3  Central Europe Early Neandertals -1.274226e-03 1.0000000
## 4  Central Europe Early Neandertals  6.257742e-04 1.0000000
## 5  Central Europe Early Neandertals  1.325774e-03 1.0000000
## 6  Central Europe Early Neandertals  1.125774e-03 1.0000000
## 7  Central Europe Early Neandertals -7.422577e-05 1.0000000
## 8  Central Europe Early Neandertals  3.257742e-04 1.0000000
## 9  Central Europe Early Neandertals  7.257742e-04 1.0000000
## 11 Central Europe  Late Neandertals -6.742258e-04 1.0000000
## 12 Central Europe  Late Neandertals  2.257742e-04 1.0000000
## 13 Central Europe  Late Neandertals -3.742258e-04 1.0000000
## 16 Central Europe  Late Neandertals -3.742258e-04 1.0000000
## 17 Western Europe  Late Neandertals -8.198511e-04 1.0000000
## 18 Western Europe  Late Neandertals -1.219851e-03 1.0000000
## 19 Western Europe Early Neandertals -3.198511e-04 1.0000000
## 20 Western Europe  Late Neandertals  9.801489e-04 1.0000000
## 21 Western Europe Early Neandertals -1.198511e-04 1.0000000
## 22 Western Europe Early Neandertals  8.014890e-05 1.0000000
## 23 Western Europe Early Neandertals -1.198511e-04 1.0000000
## 24 Western Europe  Late Neandertals -1.985110e-05 1.0000000
## 25 Western Europe  Late Neandertals  5.801489e-04 1.0000000
## 26 Western Europe  Late Neandertals -8.198511e-04 1.0000000
## 28 Western Europe  Late Neandertals -7.198511e-04 1.0000000
## 29 Western Europe  Late Neandertals  6.801489e-04 1.0000000
## 31 Western Europe Early Neandertals -4.198511e-04 1.0000000
## 32 Western Europe Early Neandertals -1.319851e-03 1.0000000
## 34 Western Europe  Late Neandertals -9.198511e-04 1.0000000
## 35 Western Europe Early Neandertals -4.198511e-04 1.0000000
## 36 Central Europe  Late Neandertals -1.074226e-03 1.0000000
## 42 Southwest Asia  Late Neandertals  4.000001e-04 1.0000000
## 43 Southwest Asia  Late Neandertals -3.999999e-04 1.0000000
## 44 Southwest Asia Early Neandertals -7.999999e-04 1.0000000
## 45 Southwest Asia Early Neandertals  8.000001e-04 1.0000000
## 46 Western Europe MIS 3         EMH  6.598921e-04 1.0000000
## 47 Western Europe MIS 3         EMH -6.611079e-04 1.0000000
## 48 Central Europe MIS 3         EMH -1.015227e-03 1.0000000
## 49 Central Europe MIS 3         EMH  6.972729e-04 1.0000000
## 51 Central Europe MIS 3         EMH -1.236727e-03 1.0000000
## 52 Western Europe MIS 2         EMH -1.346608e-03 1.0000000
## 53 Western Europe MIS 3         EMH  9.618921e-04 1.0000000
## 54 Western Europe MIS 2         EMH -2.386079e-04 1.0000000
## 57 Western Europe MIS 3         EMH -3.011079e-04 1.0000000
## 58 Western Europe MIS 3         EMH  8.803921e-04 1.0000000
## 59 Western Europe MIS 3         EMH -1.238608e-03 1.0000000
## 60 Western Europe MIS 3         EMH  2.948921e-04 1.0000000
## 61 Western Europe MIS 3         EMH  1.523921e-04 1.0000000
## 62 Southwest Asia MIS 2         EMH -4.351932e-04 1.0000000
## 63 Central Europe MIS 3         EMH -6.822271e-04 1.0000000
## 64 Central Europe MIS 3         EMH -2.177271e-04 1.0000000
## 65 Central Europe MIS 3         EMH -8.237271e-04 1.0000000
## 67 Southwest Asia MIS 5         EMH  8.948068e-04 1.0000000
## 68 Southwest Asia MIS 5         EMH -6.931947e-07 1.0000000
## 69 Southwest Asia MIS 5         EMH  1.170807e-03 1.0000000
## 70 Southwest Asia MIS 5         EMH  1.592307e-03 1.0000000
## 71 Western Europe MIS 2         EMH -9.086079e-04 1.0000000
## 72 Western Europe MIS 2         EMH -1.533108e-03 1.0000000
## 73 Southwest Asia MIS 5         EMH -6.481932e-04 1.0000000
## 75 Southwest Asia MIS 5         EMH -9.346932e-04 1.0000000
```

```
#Table 10C
preds <- data.frame(type = c(rep("Neandertals",3),rep("EMH",3)), location = c("Central Europe","Western Europe","Southwest Asia","Central Europe","Western Europe","Southwest Asia"))
preds$yhat <- predict(allModel_epLsar,newdata=preds, interval = "confidence")
preds
```

```
##          type       location    yhat.fit    yhat.lwr    yhat.upr
## 1 Neandertals Central Europe 0.003288235 0.002633469 0.003943002
## 2 Neandertals Western Europe 0.002768182 0.002192610 0.003343754
## 3 Neandertals Southwest Asia 0.003433333 0.002331197 0.004535470
## 4         EMH Central Europe 0.003198625 0.002244147 0.004153103
## 5         EMH Western Europe 0.003434321 0.002712804 0.004155839
## 6         EMH Southwest Asia 0.002927562 0.001973085 0.003882040
```

# Table 11

```
#Table 11A
########################################
##Two-way ANOVA 
########################################
allModel_Tfv <- lm(Tfv ~ location*type, data=dat)
summary(allModel_Tfv)
```

```
## 
## Call:
## lm(formula = Tfv ~ location * type, data = dat)
## 
## Residuals:
##      Min       1Q   Median       3Q      Max 
## -10132.8  -3361.5    832.8   3374.9   7494.8 
## 
## Coefficients:
##                                        Estimate Std. Error t value
## (Intercept)                             9278.37    1575.55   5.889
## locationSouthwest Asia                   854.39    2228.16   0.383
## locationWestern Europe                    29.77    1975.05   0.015
## typeNeandertals                         -607.10    1910.63  -0.318
## locationSouthwest Asia:typeNeandertals  -102.10    3072.89  -0.033
## locationWestern Europe:typeNeandertals  2723.79    2443.70   1.115
##                                        Pr(>|t|)    
## (Intercept)                            1.28e-07 ***
## locationSouthwest Asia                    0.703    
## locationWestern Europe                    0.988    
## typeNeandertals                           0.752    
## locationSouthwest Asia:typeNeandertals    0.974    
## locationWestern Europe:typeNeandertals    0.269    
## ---
## Signif. codes:  0 '***' 0.001 '**' 0.01 '*' 0.05 '.' 0.1 ' ' 1
## 
## Residual standard error: 4456 on 69 degrees of freedom
## Multiple R-squared:  0.05943,    Adjusted R-squared:  -0.00873 
## F-statistic: 0.8719 on 5 and 69 DF,  p-value: 0.5046
```

```
#Check assumptions
plot(allModel_Tfv,which=1)
```

```
plot(allModel_Tfv,which=2)
```

```
plot(allModel_Tfv,which=3)
```

```
plot(allModel_Tfv,which=4)
```

```
#Table 10B
library(MASS)
dat$vegetation <- as.character(dat$vegetation)
rrHuber <- rlm(Tfv ~ location*type, data =dat[!is.na(dat$vegetation) ,])
summary(rrHuber)
```

```
## 
## Call: rlm(formula = Tfv ~ location * type, data = dat[!is.na(dat$vegetation), 
##     ])
## Residuals:
##      Min       1Q   Median       3Q      Max 
## -10650.5  -3525.2    771.4   2978.8   7386.3 
## 
## Coefficients:
##                                        Value     Std. Error t value  
## (Intercept)                            9278.3732 1626.9853     5.7028
## locationSouthwest Asia                 1372.1555 2300.9046     0.5964
## locationWestern Europe                   71.0118 2039.5339     0.0348
## typeNeandertals                        -467.0514 1973.0094    -0.2367
## locationSouthwest Asia:typeNeandertals  404.2428 3173.2142     0.1274
## locationWestern Europe:typeNeandertals 2643.8750 2532.5473     1.0440
## 
## Residual standard error: 4839 on 68 degrees of freedom
```

```
2*(1-pnorm(abs(summary(rrHuber)$coefficient[,3])))
```

```
##                            (Intercept) 
##                           1.178545e-08 
##                 locationSouthwest Asia 
##                           5.509382e-01 
##                 locationWestern Europe 
##                           9.722251e-01 
##                        typeNeandertals 
##                           8.128738e-01 
## locationSouthwest Asia:typeNeandertals 
##                           8.986300e-01 
## locationWestern Europe:typeNeandertals 
##                           2.965045e-01
```

```
#Final Huber Weights
hweights <- data.frame(dat[!is.na(dat$vegetation),], resid = rrHuber$resid, weight = rrHuber$w)
hweights2 <- hweights[order(rrHuber$w), ]
hweights2
```

```
##                site   specimen    epLsar       Tfv vegetation
## 69           Qafzeh          7 0.0041015     0.000      Mixed
## 43           Kebara          2 0.0031000     0.000      Mixed
## 40         Shanidar          3 0.0053000  1172.640      Mixed
## 2           Krapina         49 0.0025000  1039.300     Closed
## 5           Krapina         55 0.0045000  1185.730     Closed
## 57          LesRois          3 0.0030875 16735.674       Open
## 47      Brassempouy       BR94 0.0027275  2061.425       Open
## 55          Lachaud          4 0.0055190  2165.205       Open
## 1           Krapina        131 0.0017000  4261.490     Closed
## 3           Krapina         73 0.0019000  8031.830     Closed
## 4           Krapina         75 0.0038000  9661.460     Closed
## 6           Krapina        157 0.0043000  4906.220     Closed
## 7           Krapina         70 0.0031000  5192.490     Closed
## 8           Krapina         92 0.0035000  4255.480     Closed
## 9           Krapina        130 0.0039000  9504.070     Closed
## 10          Krapina        132 0.0050000 11031.710     Closed
## 11          Vindija       12.2 0.0025000 10952.870      Mixed
## 12          Vindija       12.3 0.0034000  9812.160      Mixed
## 13          Vindija       12.4 0.0028000 13689.340      Mixed
## 14          Vindija       12.7 0.0068000 12094.760      Mixed
## 15            Kulna          1 0.0013000 13077.600      Mixed
## 16            Ochoz          1 0.0028000 14563.420      Mixed
## 17             Arcy          0 0.0018000 14132.420       Open
## 18             Arcy          0 0.0014000 15885.660       Open
## 19           Biache          1 0.0023000  8584.140     Closed
## 20            Combe          5 0.0036000 12423.390       Open
## 21            Suard          0 0.0025000 14196.790       Open
## 22               BD         10 0.0027000 11897.110       Open
## 23               BD         12 0.0025000 12113.070       Open
## 24     La Ferrassie          1 0.0026000 16331.090      Mixed
## 25     La Ferrassie          2 0.0032000 12166.670      Mixed
## 26         La Quina          5 0.0018000  6530.290       Open
## 27      Le Moustier          1 0.0005000 10308.820       Open
## 28         Le Petit          3 0.0019000  8611.000       Open
## 29         Marillac          0 0.0033000 14001.810       Open
## 31            Moula        259 0.0022000 11600.830     Closed
## 32            Moula        588 0.0013000 15069.990     Closed
## 33            Moula          0 0.0043000 10806.620     Closed
## 34      St. Cesaire          0 0.0017000  5461.790      Mixed
## 35       Pontnewydd         10 0.0022000 13234.760      Mixed
## 36         Subalyuk          1 0.0021000 14151.770       Open
## 37        Zafarraya          0 0.0054000 11348.030     Closed
## 38        Zafarraya          0 0.0054000 11041.150     Closed
## 39        Zafarraya          0 0.0056000  6304.950     Closed
## 41             Amud          1 0.0013000 13039.350      Mixed
## 42             Amud          2 0.0039000 16918.340      Mixed
## 44            Tabun          0 0.0027000 13129.820     Closed
## 45            Tabun          0 0.0043000 12281.240     Closed
## 46      Brassempouy       BR90 0.0040485 12077.535       Open
## 48   DolniVestonice         13 0.0018490 12632.026      Mixed
## 49   DolniVestonice         14 0.0035615  4848.976      Mixed
## 50   DolniVestonice         15 0.0054975  6034.072      Mixed
## 51   DolniVestonice          3 0.0016275 10577.267      Mixed
## 52       Farincourt          1 0.0020420  3556.818       Open
## 53 GrotteDesEnfants       GdE4 0.0043505  4151.129       Open
## 54         Isturitz        115 0.0031500 12411.731      Mixed
## 56          Lachaud          5 0.0051765  6438.888       Open
## 58          LesRois          5 0.0042690 11631.483       Open
## 59          LesRois          7 0.0021500 14808.913       Open
## 60          LesRois   NoNumber 0.0036835 12704.369       Open
## 61          LesRois   NoNumber 0.0035410  7315.665       Open
## 62            Ohalo          2 0.0024955 14355.555      Mixed
## 63           Pavlov          1 0.0021820 13068.702      Mixed
## 64           Pavlov         23 0.0026465  6899.535      Mixed
## 65           Pavlov         25 0.0020405  4574.831      Mixed
## 66           Pavlov    5slash1 0.0061845 15591.576      Mixed
## 67           Qafzeh          5 0.0038255 10052.726      Mixed
## 68           Qafzeh          6 0.0029300 11922.771      Mixed
## 70           Qafzeh          9 0.0045230 10628.356      Mixed
## 71      RoundDuBary         RB 0.0024800 12740.693       Open
## 72     SaintGermain 1970-07-12 0.0018555 11514.502       Open
## 73            Skhul          2 0.0022825 13033.947      Mixed
## 74            Skhul          4 0.0012665  5765.140      Mixed
## 75            Skhul          5 0.0019960 15303.622      Mixed
##          location  time        type        resid    weight
## 69 Southwest Asia MIS 5         EMH -10650.52863 0.6110885
## 43 Southwest Asia  Late Neandertals -10587.72011 0.6147597
## 40 Southwest Asia  Late Neandertals  -9415.08011 0.6913341
## 2  Central Europe Early Neandertals  -7772.02181 0.8374167
## 5  Central Europe Early Neandertals  -7625.59181 0.8534972
## 57 Western Europe MIS 3         EMH   7386.28934 0.8811427
## 47 Western Europe MIS 3         EMH  -7287.95994 0.8930391
## 55 Western Europe MIS 2         EMH  -7184.18036 0.9059396
## 1  Central Europe Early Neandertals  -4549.83181 1.0000000
## 3  Central Europe Early Neandertals   -779.49181 1.0000000
## 4  Central Europe Early Neandertals    850.13819 1.0000000
## 6  Central Europe Early Neandertals  -3905.10181 1.0000000
## 7  Central Europe Early Neandertals  -3618.83181 1.0000000
## 8  Central Europe Early Neandertals  -4555.84181 1.0000000
## 9  Central Europe Early Neandertals    692.74819 1.0000000
## 10 Central Europe Early Neandertals   2220.38819 1.0000000
## 11 Central Europe  Late Neandertals   2141.54819 1.0000000
## 12 Central Europe  Late Neandertals   1000.83819 1.0000000
## 13 Central Europe  Late Neandertals   4878.01819 1.0000000
## 14 Central Europe  Late Neandertals   3283.43819 1.0000000
## 15 Central Europe  Late Neandertals   4266.27819 1.0000000
## 16 Central Europe  Late Neandertals   5752.09819 1.0000000
## 17 Western Europe  Late Neandertals   2606.21143 1.0000000
## 18 Western Europe  Late Neandertals   4359.45143 1.0000000
## 19 Western Europe Early Neandertals  -2942.06857 1.0000000
## 20 Western Europe  Late Neandertals    897.18143 1.0000000
## 21 Western Europe Early Neandertals   2670.58143 1.0000000
## 22 Western Europe Early Neandertals    370.90143 1.0000000
## 23 Western Europe Early Neandertals    586.86143 1.0000000
## 24 Western Europe  Late Neandertals   4804.88143 1.0000000
## 25 Western Europe  Late Neandertals    640.46143 1.0000000
## 26 Western Europe  Late Neandertals  -4995.91857 1.0000000
## 27 Western Europe  Late Neandertals  -1217.38857 1.0000000
## 28 Western Europe  Late Neandertals  -2915.20857 1.0000000
## 29 Western Europe  Late Neandertals   2475.60143 1.0000000
## 31 Western Europe Early Neandertals     74.62143 1.0000000
## 32 Western Europe Early Neandertals   3543.78143 1.0000000
## 33 Western Europe Early Neandertals   -719.58857 1.0000000
## 34 Western Europe  Late Neandertals  -6064.41857 1.0000000
## 35 Western Europe Early Neandertals   1708.55143 1.0000000
## 36 Central Europe  Late Neandertals   5340.44819 1.0000000
## 37 Western Europe  Late Neandertals   -178.17857 1.0000000
## 38 Western Europe  Late Neandertals   -485.05857 1.0000000
## 39 Western Europe  Late Neandertals  -5221.25857 1.0000000
## 41 Southwest Asia  Late Neandertals   2451.62989 1.0000000
## 42 Southwest Asia  Late Neandertals   6330.61989 1.0000000
## 44 Southwest Asia Early Neandertals   2542.09989 1.0000000
## 45 Southwest Asia Early Neandertals   1693.51989 1.0000000
## 46 Western Europe MIS 3         EMH   2728.15035 1.0000000
## 48 Central Europe MIS 3         EMH   3353.65254 1.0000000
## 49 Central Europe MIS 3         EMH  -4429.39724 1.0000000
## 50 Central Europe MIS 3         EMH  -3244.30135 1.0000000
## 51 Central Europe MIS 3         EMH   1298.89399 1.0000000
## 52 Western Europe MIS 2         EMH  -5792.56650 1.0000000
## 53 Western Europe MIS 3         EMH  -5198.25615 1.0000000
## 54 Western Europe MIS 2         EMH   3062.34614 1.0000000
## 56 Western Europe MIS 2         EMH  -2910.49740 1.0000000
## 58 Western Europe MIS 3         EMH   2282.09806 1.0000000
## 59 Western Europe MIS 3         EMH   5459.52838 1.0000000
## 60 Western Europe MIS 3         EMH   3354.98436 1.0000000
## 61 Western Europe MIS 3         EMH  -2033.72013 1.0000000
## 62 Southwest Asia MIS 2         EMH   3705.02642 1.0000000
## 63 Central Europe MIS 3         EMH   3790.32924 1.0000000
## 64 Central Europe MIS 3         EMH  -2378.83854 1.0000000
## 65 Central Europe MIS 3         EMH  -4703.54194 1.0000000
## 66 Central Europe MIS 3         EMH   6313.20330 1.0000000
## 67 Southwest Asia MIS 5         EMH   -597.80304 1.0000000
## 68 Southwest Asia MIS 5         EMH   1272.24282 1.0000000
## 70 Southwest Asia MIS 5         EMH    -22.17280 1.0000000
## 71 Western Europe MIS 2         EMH   3391.30800 1.0000000
## 72 Western Europe MIS 2         EMH   2165.11678 1.0000000
## 73 Southwest Asia MIS 5         EMH   2383.41812 1.0000000
## 74 Southwest Asia MIS 5         EMH  -4885.38875 1.0000000
## 75 Southwest Asia MIS 5         EMH   4653.09307 1.0000000
```

```
#Table 10C
preds <- data.frame(type = c(rep("Neandertals",3),rep("EMH",3)), location = c("Central Europe","Western Europe","Southwest Asia","Central Europe","Western Europe","Southwest Asia"))
preds$yhat <- predict(allModel_Tfv,newdata=preds, interval = "confidence")
preds
```

```
##          type       location  yhat.fit  yhat.lwr  yhat.upr
## 1 Neandertals Central Europe  8671.276  6515.108 10827.445
## 2 Neandertals Western Europe 11424.836  9529.458 13320.215
## 3 Neandertals Southwest Asia  9423.565  5794.192 13052.938
## 4         EMH Central Europe  9278.373  6135.244 12421.503
## 5         EMH Western Europe  9308.145  6932.163 11684.127
## 6         EMH Southwest Asia 10132.765  6989.635 13275.894
```

Table 12

```
#Table 12A
#Testing if the distributions are signifcantly different 
ks.test(dat$epLsar[dat$type=="EMH"],dat$epLsar[dat$type=="Neandertals"],exact=FALSE)
```

```
## Warning in ks.test(dat$epLsar[dat$type == "EMH"], dat$epLsar[dat$type == :
## p-value will be approximate in the presence of ties
```

```
## 
##  Two-sample Kolmogorov-Smirnov test
## 
## data:  dat$epLsar[dat$type == "EMH"] and dat$epLsar[dat$type == "Neandertals"]
## D = 0.13333, p-value = 0.9062
## alternative hypothesis: two-sided
```

```
ks.test(dat$Tfv[dat$type=="EMH"],dat$Tfv[dat$type=="Neandertals"],exact=FALSE)
```

```
## Warning in ks.test(dat$Tfv[dat$type == "EMH"], dat$Tfv[dat$type ==
## "Neandertals"], : p-value will be approximate in the presence of ties
```

```
## 
##  Two-sample Kolmogorov-Smirnov test
## 
## data:  dat$Tfv[dat$type == "EMH"] and dat$Tfv[dat$type == "Neandertals"]
## D = 0.15556, p-value = 0.7764
## alternative hypothesis: two-sided
```

```
#Table 12B
ks.test(dat$epLsar[dat$type=="EMH" & dat$vegetation=="Open"],dat$epLsar[dat$type=="Neandertals" & dat$vegetation=="Open"],exact=FALSE)
```

```
## Warning in ks.test(dat$epLsar[dat$type == "EMH" & dat$vegetation ==
## "Open"], : p-value will be approximate in the presence of ties
```

```
## 
##  Two-sample Kolmogorov-Smirnov test
## 
## data:  dat$epLsar[dat$type == "EMH" & dat$vegetation == "Open"] and dat$epLsar[dat$type == "Neandertals" & dat$vegetation == "Open"]
## D = 0.51049, p-value = 0.0896
## alternative hypothesis: two-sided
```

```
ks.test(dat$Tfv[dat$type=="EMH" & dat$vegetation=="Open"],dat$Tfv[dat$type=="Neandertals" & dat$vegetation=="Open"],exact=FALSE)
```

```
## 
##  Two-sample Kolmogorov-Smirnov test
## 
## data:  dat$Tfv[dat$type == "EMH" & dat$vegetation == "Open"] and dat$Tfv[dat$type == "Neandertals" & dat$vegetation == "Open"]
## D = 0.38462, p-value = 0.3414
## alternative hypothesis: two-sided
```

```
ks.test(dat$epLsar[dat$type=="EMH" & dat$vegetation=="Mixed"],dat$epLsar[dat$type=="Neandertals" & dat$vegetation=="Mixed"],exact=FALSE)
```

```
## Warning in ks.test(dat$epLsar[dat$type == "EMH" & dat$vegetation ==
## "Mixed"], : p-value will be approximate in the presence of ties
```

```
## 
##  Two-sample Kolmogorov-Smirnov test
## 
## data:  dat$epLsar[dat$type == "EMH" & dat$vegetation == "Mixed"] and dat$epLsar[dat$type == "Neandertals" & dat$vegetation == "Mixed"]
## D = 0.18487, p-value = 0.9556
## alternative hypothesis: two-sided
```

```
ks.test(dat$Tfv[dat$type=="EMH" & dat$vegetation=="Mixed"],dat$Tfv[dat$type=="Neandertals" & dat$vegetation=="Mixed"],exact=FALSE)
```

```
## Warning in ks.test(dat$Tfv[dat$type == "EMH" & dat$vegetation ==
## "Mixed"], : p-value will be approximate in the presence of ties
```

```
## 
##  Two-sample Kolmogorov-Smirnov test
## 
## data:  dat$Tfv[dat$type == "EMH" & dat$vegetation == "Mixed"] and dat$Tfv[dat$type == "Neandertals" & dat$vegetation == "Mixed"]
## D = 0.26471, p-value = 0.6551
## alternative hypothesis: two-sided
```

```
ks.test(dat$Tfv[dat$type=="EMH" & dat$location=="Western Europe"],dat$Tfv[dat$type=="Neandertals" & dat$location=="Western Europe"],exact=FALSE)
```

```
## 
##  Two-sample Kolmogorov-Smirnov test
## 
## data:  dat$Tfv[dat$type == "EMH" & dat$location == "Western Europe"] and dat$Tfv[dat$type == "Neandertals" & dat$location == "Western Europe"]
## D = 0.29221, p-value = 0.4582
## alternative hypothesis: two-sided
```

```
ks.test(dat$Tfv[dat$type=="EMH" & dat$location=="Central Europe"],dat$Tfv[dat$type=="Neandertals" & dat$location=="Central Europe"],exact=FALSE)
```

```
## 
##  Two-sample Kolmogorov-Smirnov test
## 
## data:  dat$Tfv[dat$type == "EMH" & dat$location == "Central Europe"] and dat$Tfv[dat$type == "Neandertals" & dat$location == "Central Europe"]
## D = 0.23529, p-value = 0.924
## alternative hypothesis: two-sided
```

```
ks.test(dat$Tfv[dat$type=="EMH" & dat$location=="Southwest Asia"],dat$Tfv[dat$type=="Neandertals" & dat$location=="Southwest Asia"],exact=FALSE)
```

```
## Warning in ks.test(dat$Tfv[dat$type == "EMH" & dat$location == "Southwest
## Asia"], : p-value will be approximate in the presence of ties
```

```
## 
##  Two-sample Kolmogorov-Smirnov test
## 
## data:  dat$Tfv[dat$type == "EMH" & dat$location == "Southwest Asia"] and dat$Tfv[dat$type == "Neandertals" & dat$location == "Southwest Asia"]
## D = 0.29167, p-value = 0.9324
## alternative hypothesis: two-sided
```

```
ks.test(dat$epLsar[dat$type=="EMH" & dat$location=="Western Europe"],dat$epLsar[dat$type=="Neandertals" & dat$location=="Western Europe"],exact=FALSE)
```

```
## Warning in ks.test(dat$epLsar[dat$type == "EMH" & dat$location == "Western
## Europe"], : p-value will be approximate in the presence of ties
```

```
## 
##  Two-sample Kolmogorov-Smirnov test
## 
## data:  dat$epLsar[dat$type == "EMH" & dat$location == "Western Europe"] and dat$epLsar[dat$type == "Neandertals" & dat$location == "Western Europe"]
## D = 0.3961, p-value = 0.1364
## alternative hypothesis: two-sided
```

```
ks.test(dat$epLsar[dat$type=="EMH" & dat$location=="Central Europe"],dat$epLsar[dat$type=="Neandertals" & dat$location=="Central Europe"],exact=FALSE)
```

```
## Warning in ks.test(dat$epLsar[dat$type == "EMH" & dat$location == "Central
## Europe"], : p-value will be approximate in the presence of ties
```

```
## 
##  Two-sample Kolmogorov-Smirnov test
## 
## data:  dat$epLsar[dat$type == "EMH" & dat$location == "Central Europe"] and dat$epLsar[dat$type == "Neandertals" & dat$location == "Central Europe"]
## D = 0.27206, p-value = 0.8155
## alternative hypothesis: two-sided
```

```
ks.test(dat$epLsar[dat$type=="EMH" & dat$location=="Southwest Asia"],dat$epLsar[dat$type=="Neandertals" & dat$location=="Southwest Asia"],exact=FALSE)
```

```
## 
##  Two-sample Kolmogorov-Smirnov test
## 
## data:  dat$epLsar[dat$type == "EMH" & dat$location == "Southwest Asia"] and dat$epLsar[dat$type == "Neandertals" & dat$location == "Southwest Asia"]
## D = 0.33333, p-value = 0.8407
## alternative hypothesis: two-sided
```

# Figures

```
dat <- read.csv("/Users/gregorymatthews/Dropbox/kruegerTeeth/modern_human_data.csv")[,1:3]

dat$Group <- as.character(dat$Group)
dat$Group[dat$Group=="Coast Tsimshian"] <- "Tsimshian"
dat$Group <- factor(dat$Group)

library(ggplot2)
bioarc <- setdiff(levels(dat$Group), c("EMH","Neandertals"))

g <- list()
for (i in 1:length(bioarc)){

temp <- subset(dat,Group %in% c(bioarc[i],"EMH","Neandertals"))
temp$Group <- factor(temp$Group, levels = c(bioarc[i],"EMH","Neandertals"))

g[[i]] <- ggplot(aes(x = Tfv, y = epLsar, colour = Group), data = temp) + geom_point() + stat_ellipse(size = 2, alpha = 0.9) + scale_colour_manual(values = c("red","blue","green")) + xlim(-10000,22000) + ylim(-0.002,0.008) + ggtitle(bioarc[i]) + theme(legend.position = "none")
print(g[[i]]) 

}
```

```
temp <- subset(dat,Group %in% c("EMH","Neandertals"))
temp$Group <- factor(temp$Group, levels = c("EMH","Neandertals"))

h <- ggplot(aes(x = Tfv, y = epLsar, colour = Group), data = temp) + geom_point() + stat_ellipse(size = 2, alpha = 0.9) + scale_colour_manual(values = c("blue","green")) + xlim(-10000,22000) + ylim(-0.002,0.008) + ggtitle("EMH vs. Neandertal") 
print(h)
```

```
tiff("/Users/gregorymatthews/Dropbox/kruegerTeeth/new_figure_2.tiff", units = "in", w = 8.5, h = 11, res = 500)
library(gridExtra)
```

```
## 
## Attaching package: 'gridExtra'
```

```
## The following object is masked from 'package:dplyr':
## 
##     combine
```

```
grid.arrange(h,g[[1]],g[[2]],g[[3]],g[[4]],g[[5]],g[[6]],g[[7]],nrow = 4)
dev.off()
```

```
## quartz_off_screen 
##                 2
```

```
ggplot(aes(x = Tfv), data = dat) + geom_density()
```

```
ggplot(aes(x = epLsar), data = dat) + geom_density()
```
