# Supplementary material for: Anterior tooth-use behaviors among early modern humans and Neandertals
Source: PLoS One. 2019 Nov 27;14(11):e0224573. doi: 10.1371/journal.pone.0224573 (PMC6880970; doi:10.1371/journal.pone.0224573)
Supplement: Attachment — Submitted filename: Krueger et al._R2_RESPONSE TO REVIEWERS.pdf [file pone.0224573.s006.pdf]

LOYOLA
UNIVERSITY CHICAGO

Department of Anthropology
Lake Shore Campus
1032 W. Sheridan Road | Chicago, Illinois 60660
Phone 773.508.3485 | Fax 773.508.3295

October 15, 2019

Dear Editor Pinhasi,

Please find our research article resubmission (R2), *Anterior tooth-use behaviors among early modern humans and Neandertals*, for consideration in PLoS ONE.

Below please find responses to the reviewers' concerns in blue. We thank the reviewers for their time, effort, and constructive comments.

All my best,

Dr. Kristin L. Krueger
Associate Professor of Anthropology

Review of Krueger et al. for PONE:
The authors have successfully responded to the critiques and suggestions of the reviewers. The result is an improved manuscript that is nearly ready for publication. The comments below are meant to further improve this excellent analysis and comparison of Middle and Upper Paleolithic anterior tooth texture. Although the relationship between anterior tooth use and cultural inferiority / superiority is not immediately obvious, in the Discussion the authors explain better how perceptions of Neandertals can influence the explanation of the variation in tooth wear and other studies. The lack of differences imply that similar behaviors were performed by Middle and Upper Paleolithic peoples and some of these were mimicked by their Holocene counterparts. The paper will have a high impact on the field and I encourage the rapid publication of the manuscript.
Specific comments:
Introduction

- "had a more complex division of labor for resource acquisition" doesn't quite make sense. More complex than whom? Maybe omit "more" "more" removed.

- "show distinctions between Neandertals and early modern humans – albeit, with some overlap" —consider revising, or perhaps replace with statistically meaningful terms such as "show a pattern in the distinctions in the mean values for Neandertal and early modern humans, but with large standard deviations resulting in nonsignificant differences…" We have revised this

section to be more statistically meaningful. Please see lines 133-137. We also added in lines 137-145 about dentin exposure to help buttress the data about enamel wear.

M&M

- Table 2: Just double checking: you investigated La Quina 1, not La Quina 5, correct? In any event, it might be interesting to eventually examine the incisal microtexture of La Quina 5.

We investigated La Quina 5; we apologize for any confusion here. Please see the S1 supplementary data file for more information.

- "under the auspices of three attainable factors:" is unclear—consider changing to "using three factors" We changed this per the reviewer's suggestion.

- Change "environmental reconstructions, dating techniques, etc" to "environmental reconstructions and dating techniques" We changed this per the reviewer's suggestion.

- "[S1 file and SOM in 37; however, these limitations resulted in broad categories. We recognize that other researchers may use different groupings [101, 102]." Is problematic. The first clause is not a complete sentence yet includes a period and the end misses a double bracket. I recommend changing it to "as shown in the S1 file [and SOM in 37]; however, these limitations resulted in broad categories [cf. 101, 102]." We changed this per the reviewer's suggestion.

- "Underpinnings" is unclear: Change "their mathematical underpinnings are described in Scott et al." to "their mathematical descriptions are detailed in Scott et al." We changed this per the reviewer's suggestion.

- To increase clarity, maybe replace "time), six combinations in total, a" with "time)--six combinations in total—a" We changed this per the reviewer's suggestion.

Results

- Just a recommendation—the authors might want to comb through the paper carefully and omit redundancy. For example, in the Results, I felt like I read that "Closed" habitat is removed in this analysis as no early modern humans analyzed here lived in "closed" environments." or something like it multiple times in the text and captions—maybe remove one of them? There are a few others that are repeated once too often –maybe remove from table and figure captions? We changed this per the reviewer's suggestion. Thank you for noticing the redundancy!

Discussion and conclusion

- I'd recommend removing entirely "While comparisons with the Neandertal sample will be addressed in more detail below, the similarity with the Point Hope Tigara commands consideration." We removed this per the reviewer's suggestion.

- Consider changing "in European Upper Paleolithic archaeological sites and those taxa used ethnographically for the fur, hide, sinew, and other raw materials" to "in European Upper

Paleolithic archaeological sites and those taxa reported in the ethnographic record as sources of the fur, hide, sinew and other raw materials" We changed this per the reviewer's suggestion.

- "Sadlermiuuit (Table 4).: still retains track-changes and later on lines 429, 430 We corrected these errors.

- Lines 439-444--I think what we are seeing here in terms of the similarity of textures is the *eat what you can find* phenomenon of Ofar Bar-Yosef and others. Agreed!

- Perhaps change "not as behaviorally distinct as once thought." To "not as behaviorally distinct as previously considered." Or something else—"once thought." Is unclear. We changed this per the reviewer's suggestion.

This reviewer would like to thank the first author and Greg Mathews for assuaging my previous concerns about multiple groups in the values for Tfv and epLsar by doing the density plots with kernel density estimates to estimate probability and with histograms to clarify the results. The experiment demonstrates the excellent sample sizes allowing for a statistical treatment of data that showed patterns based on individual values as nonsigificant. The additional analysis improves my confidence in the validity of the results.

We thank the reviewer for providing the idea in the first place. We were happy to examine the data further to see if anything significant was missed.

Reviewer #2: The authors have resubmitted a revised version of their manuscript, titled "Anterior tooth-use behaviors among early modern humans and Neandertals", but unfortunately most of the reviewers' comments were not taken into account.

We did not add much of this reviewer's comments in the manuscript; however, that does not mean the comments were neither meaningful nor impactful. Indeed, these comments forced us to think about our data using a biomechanical perspective, and if that framework should be added to the discussion at hand. We ultimately decided that adding in a review section outlining both biomechanical and comparative approaches was a better fit for the direction of this paper.

In my opinion the response to reviewers included in the rebuttal letter is not really sufficient to address major criticisms of their work. Specifically, I find very odd to discuss about Neanderthal anterior tooth wear, culture and bite force without even mentioning the anterior dental loading hypothesis. The fact that in other Neanderthal anterior tooth-use studies there was no mention the biomechanics of Neanderthal anterior tooth wear, it is not a valid excuse to ignore this important aspect, which indirectly it is strictly associated with cultural habits in this human species.

We thoroughly addressed this reviewer's concerns in the "response to reviewers" letter; however, we appreciate the time and effort this reviewer spent with this manuscript again. As such, we have included a new section in the Introduction titled "Biomechanical versus Comparative Approach," which details the Anterior Dental Loading Hypothesis, challenges of such work, comparative approaches, and limitations of our work. We hope that this will reflect a good faith effort in showing Reviewer 2 that we have, indeed, chosen the best framework for our data.

Moreover, a biomechanical approach focuses on the *potential* for Neandertals to produce (or modern humans to not produce) a high or heavy anterior bite force. Our data show that regardless of the *potential* for such behaviors, both hominins were *actually* performing these behaviors. These data do not provide any indication of whether the unique craniofacial traits found on Neandertals reflect an adaptation or a neutral evolutionary force, such as genetic drift, so to couch our discussion in such a framework is inappropriate. We understand the reviewer may not agree, but we are confident in this assessment of our data.

Overall, I still feel that the manuscript is largely incomplete, in terms of background information, interpretation, discussion of the results and literature review. The authors also did not acknowledge any of the limitations of their study. While I agree with the authors that the sample size is good for a paleoanthropological study, it is never ideal from a statistical point of view. I think it is always worth being cautions when interpreting the results from the analysis of relatively small fossil samples. Generally, a sentence about the limitation of the study at the end of the discussion is sufficient.

We added in limitations of direct, comparative approaches, including our study, in lines 187-189.

Finally, as I have mentioned in my previous review, the authors did not highlight enough what is new and what is not new in their study.

We respectively disagree with this assessment once again. There has never been a study that analyzes dental microwear as objectively as the method used here. Moreover, the bioarchaeological baseline used here provides a comprehensive framework for interpreting the *actual* behaviors of early modern humans versus those of their Neandertal counterparts. This study offers an innovative and unique lens into the lives of these hominins.
