# Supplementary material for: Anterior tooth-use behaviors among early modern humans and Neandertals
Source: PLoS One. 2019 Nov 27;14(11):e0224573. doi: 10.1371/journal.pone.0224573 (PMC6880970; doi:10.1371/journal.pone.0224573)
Supplement: Attachment — Submitted filename: Krueger et al. PLoS.docx [file pone.0224573.s003.docx]

This is a bold paper that seeks to compare early modern humans, Neandertals and seven bioarchaeology groups using two measures of enamel surface texture rendered from dental microwear texture analysis. The use of the incisors to unlock the behavioral repertoire of fossil hominins is provocative and the authors use an innovative approach—texture analysis coupled with an elegant statistical treatment of this exceptional dataset—to reconstruct paramasticatory actions relating to food and material processing. Although behavioral superiority is not necessarily directly tested by the incisal microwear textures, the results suggest that early modern humans and Neandertals utilized their incisors in a similar manner and the closest bioarchaeological approximation is the Point Hope Tigara and the Nunavut Territory Sadlermiut. The paper is profound in its implications backed up by enviable samples, both fossil and bioarchaeological, and I fully endorse the publication of this submission after minor revisions.

Regarding the results, the data graphs are a bit challenging to decipher—consider, if possible, demoting the bioarchaeology populations to thin dark blue ellipse lines and leave the two fossil samples as they are now, thicker lines of different brighter colors. This might add clarity to the main focus of the paper—the comparison between the two fossil hominins.

Although there are no significant differences between groups, when looking at Table 4, it seems to me that for Tfv, there are three groupings. A heavily incised group includes (1) early modern humans, Neandertals and Nunavut; (2) a medium values group includes Tigara, Puye Pueblo and Prince Rupert Tsimshian; and (3) a low values group comprising Andaman Islanders, Arikara and Island Chumash. What I would say is that recent humans can be divided into three groups but the fossils are exclusively found in the high value group, the only equivalent might be the Nunavut of the Arctic, and possibly the Tigara. For epLsar, there are two groups in my view. One group would include Nunavut and PR Tsimshian with low values and the other group would include everyone else with more elevated values.

The authors might want to consider briefly acknowledging the subjectivity of the groups. For example, Western Europe (mostly France) is quite far from Central Europe but the distance is not nearly as far as it is to SW Asia, so these groups are not necessarily equivalent in terms of location. The ecogeography would also differ between continental Europe and the near East. It might just as easily be possible to combine the Western and Central Europe groupings into one and compare this group to SW Asia. The same holds true for the habitat categories. These are minor points, however, and should be taken as suggestions for improving an already excellent paper.

Specific comments:

Title: change “overlapping” to “comparison of” or remove and shorten title

Line 62: consider citing Power and Williams (2018) at the end of the sentence and introduce food processing into the sentence: “improved food storage and processing capabilities.”

70: replace “this hominin” with “Neandertal”

84: change punctuation to “them.”

94: remove “bodies—specifically”

95: remove dash

110: replace “variations” with “variation”

116: change “showed” to “show” to agree with the verb tense of the rest of this section

120: consider removing the first sentence containing “from the dialogue” for clarity

121: In the abstract, the authors use EMH, but switch to “early modern humans” for the rest of the paper—I like the latter much better than the abbreviation—the authors may want to replace “EMH” with “early modern humans” in the abstract and elsewhere

127-8: Many early modern humans from Europe and SW Asia also exhibit shoveling

129: “average early modern human” seems vague—be more specific here

139: don’t need to mention the abbreviation “LSAMAT” since you only mention it once

147: replace “expound” with “identify”

151: remove “focal” to avoid confusion with focal animal sampling or ethnographic usages (e.g., Hewlett, 1991, Intimate Fathers)

Table 2: consider replacing “time” with “MIS” as it will increase transparency

166: “own unique” seems redundant

166-172: consider removing this paragraph, except the last half of the last sentence (see below)

172-174: Start next paragraph with “The fossil sample was examined by habitat, location and time interval.”—this would be the first sentence

192: change punctuation for “open,” “mixed,”

178-190: perhaps provide a stronger justification for the category of “mixed” as a wide range of habitats are considered

203-204: improve justification for the lumping of MIS 7-5 and MIS 4-3 into distinct groups, such as ecology or climate extremes

351: change “possible” to “possibly”

352: remove sentence beginning with “Each analysis will be…”

366: Remove “examining it through the lens of”

416: Change “was” to “were”

417: insert “dental” between “extensive” and “clamping”

426: replace “conservative” with “parsimonious”

428: replace “indicates” with “indicate” because it’s modifying “data” (plural)

436: consider removing the paragraph beginning on 436 to avoid redundancy

447: Combine “Discussion and Conclusions” into a single section and remove conclusion paragraph beginning on 447 to avoid redundancy

Literature

Power RC, Williams FL (2018) The increasing intensity of food processing during the Upper Paleolithic of western Eurasia. *Journal of Paleolithic Archaeology* 1:281–301. <http://dx.doi.org/10.1007/s41982-018-0014-x>.
