# Supplementary material for: Anterior tooth-use behaviors among early modern humans and Neandertals
Source: PLoS One. 2019 Nov 27;14(11):e0224573. doi: 10.1371/journal.pone.0224573 (PMC6880970; doi:10.1371/journal.pone.0224573)
Supplement: Attachment — Submitted filename: Krueger et al. PLoS version2.docx [file pone.0224573.s005.docx]

Review of Krueger et al. for PONE:

The authors have successfully responded to the critiques and suggestions of the reviewers. The result is an improved manuscript that is nearly ready for publication. The comments below are meant to further improve this excellent analysis and comparison of Middle and Upper Paleolithic anterior tooth texture. Although the relationship between anterior tooth use and cultural inferiority / superiority is not immediately obvious, in the Discussion the authors explain better how perceptions of Neandertals can influence the explanation of the variation in tooth wear and other studies. The lack of differences imply that similar behaviors were performed by Middle and Upper Paleolithic peoples and some of these were mimicked by their Holocene counterparts. The paper will have a high impact on the field and I encourage the rapid publication of the manuscript.

Specific comments:

Introduction

- “had a more complex division of labor for resource acquisition” doesn’t quite make sense. More complex than whom? Maybe omit “more”
- “show distinctions between Neandertals and early modern humans – albeit, with some overlap” —consider revising, or perhaps replace with statistically meaningful terms such as “show a pattern in the distinctions in the mean values for Neandertal and early modern humans, but with large standard deviations resulting in nonsignificant differences…”

M&M

- Table 2: Just double checking: you investigated La Quina 1, not La Quina 5, correct? In any event, it might be interesting to eventually examine the incisal microtexture of La Quina 5.
- “under the auspices of three attainable factors:” is unclear—consider changing to “using three factors”
- Change “environmental reconstructions, dating techniques, etc” to “environmental reconstructions and dating techniques”
- “[S1 file and SOM in 37; however, these limitations resulted in broad categories. We recognize that other researchers may use different groupings [101, 102].” Is problematic. The first clause is not a complete sentence yet includes a period and the end misses a double bracket. I recommend changing it to “as shown in the S1 file [and SOM in 37]; however, these limitations resulted in broad categories [cf. 101, 102].”
- “Underpinnings” is unclear: Change “their mathematical underpinnings are described in Scott et al.” to “their mathematical descriptions are detailed in Scott et al.”
- To increase clarity, maybe replace “time), six combinations in total, a” with “time)--six combinations in total—a”

Results

- Just a recommendation—the authors might want to comb through the paper carefully and omit redundancy. For example, in the Results, I felt like l read that “Closed” habitat is removed in this analysis as no early modern humans analyzed here lived in “closed” environments.” or something like it multiple times in the text and captions—maybe remove one of them? There are a few others that are repeated once too often –maybe remove from table and figure captions?

Discussion and conclusion

- I’d recommend removing entirely “While comparisons with the Neandertal sample will be addressed in more detail below, the similarity with the Point Hope Tigara commands consideration.”
- Consider changing “in European Upper Paleolithic archaeological sites and those taxa used ethnographically for the fur, hide, sinew, and other raw materials” to “in European Upper Paleolithic archaeological sites and those taxa reported in the ethnographic record as sources of the fur, hide, sinew and other raw materials”
- “Sadlermiuuit (Table 4).: still retains track-changes and later on lines 429, 430
- Lines 439-444--I think what we are seeing here in terms of the similarity of textures is the *eat what you can find* phenomenon of Ofar Bar-Yosef and others.
- Perhaps change “not as behaviorally distinct as once thought.” To “not as behaviorally distinct as previously considered.” Or something else—“once thought.” Is unclear.

This reviewer would like to thank the first author and Greg Mathews for assuaging my previous concerns about multiple groups in the values for Tfv and epLsar by doing the density plots with kernel density estimates to estimate probability and with histograms to clarify the results. The experiment demonstrates the excellent sample sizes allowing for a statistical treatment of data that showed patterns based on individual values as nonsigificant. The additional analysis improves my confidence in the validity of the results.
